# Comparison of gene expression in the red imported fire ant (*Solenopsis invicta*) under different temperature conditions

**DOI:** 10.1038/s41598-021-95779-w

**Published:** 2021-08-13

**Authors:** Mohammad Vatanparast, Robert T. Puckett, Deuk-Soo Choi, Youngjin Park

**Affiliations:** 1 Plant Quarantine Technology Center, Animal and Plant Quarantine Agency, Gimcheon, 39660 Republic of Korea; 2 Department of Entomology, Texas A&M University, College Station, TX 77843 USA

**Keywords:** Entomology, Gene ontology

## Abstract

The red imported fire ant (RIFA), *Solenopsis invicta* Buren is native to South America and is known as a global problematic invasive species. This study focused on the molecular response of RIFA by comparing gene expression profiles after exposing ants to low (10 °C) and high (40 °C) temperature stress and comparing them to untreated controls (30 °C). A total of 99,085 unigenes (the clustered non-redundant transcripts that are filtered from the longest assembled contigs) were obtained, of which 19,154 were annotated with gene descriptions, gene ontology terms, and metabolic pathways. 86 gene ontology (GO) functional sub-groups and 23 EggNOG terms resulted. Differentially expressed genes (DEGs) with log_2_FC ≥ 10 were screened and were compared at different temperatures. We found 203, 48, and 66 specific DEGs co-regulated at 10, 20, and 40 °C. Comparing transcriptome profiles for differential gene expression resulted in various DE genes, including cytochrome P450, NADH dehydrogenase subunit 1, cuticle protein and heat shock protein (HSP), which have previously been reported to be involved in cold and high temperature resistance. GO analysis revealed that antioxidant activity is up-regulated under high temperature stress. We verified the RNA-seq data by qPCR on 20 up- and down-regulated DEGs. These findings provide a basis for future understanding of the adaptation mechanisms of RIFA and the molecular mechanisms underlying the response to low and high temperatures.

## Introduction

The red imported fire ant (RIFA), *Solenopsis invicta* Buren (Hymenoptera: Formicidae), is a global invasive and aggressive species native to South America that is considered one of the world's top 100 invasive pests^1,2^. RIFA was first recorded in the United States in the 1930s, and it has since spread to other temperate regions around the world^1^. *S. invicta* populations are now established in the United States, Mexico, Australia, New Zealand, China, Malaysia, Japan, Singapore, and the West Indies^3,4^. It is classified as a quarantined pest in Korea. In their invasive range, RIFA has been shown to have negative effects on human health, public safety, habitats, agriculture, and native biodiversity^5^. RIFA invasions have the potential to endanger 41 species on China's National List of Protected Wildlife, including 22 birds, one amphibian, and 18 reptiles, as well as cause resource constraints on arthropod populations^6^.

Temperature is one of the most important abiotic factors that influences insect distribution and life history^7,8^. Temperatures affect insect population dynamics and geographic distribution by interfering with metabolic processes such as alimentation, digestion, detoxification, mating, and growth^9–13^. Extreme temperatures pose a threat to the stability of insect populations and can have a negative impact on their development. RIFA shows ecological adaptability to exceptionally high temperatures in warm areas, but cold temperatures have a direct effect on its regional distribution^14^. It tends to build earthen mounds in open, sunny areas to regulate the temperature of its brood^15^ and is scarce in heavily forested areas^16^. Some tropical areas that are warm and moist enough to sustain *S. invicta* are heavily forested, making them unsuitable for fire ant habitat. However, because of this species' ability to colonize disturbed areas quickly (and move soil or plant material), any deforested areas are at risk^16^. If the native ant fauna is sufficiently resistant, *S. invicta* may be unable to invade certain areas. Because of various natural enemies in South America, *S. invicta* is thought to be more widespread in the United States than in its native South America^17^. In a preliminary study, The CLIMEX model was used to determine the risks of RIFA establishing in Oceania. According to that paper, the fire ant could develop in much cooler climates than those classified as “possible” before^18^. It has been well documented that temperature indices provide a useful predictive tool for predicting the potential distribution of RIFA in newly invaded systems^19^. RIFA is of great concern in China, where studies have been conducted to determine its tolerance to extreme temperatures in order to predict its potential range expansion^14^. Since climate change has raised the risk of invasion, South Korea is a new region to apply CLIMEX to anticipate the probable distribution of invasive pests^20^. In South Korea, a machine-learning-based statistical approach was utilized to estimate the potential distribution of the red imported fire ant, but this study only employed distribution data^21^. In another study of the invasive risk of RIFA in South Korea, other elements, such as climatic adaptability, geographical characteristics, and the impact of agricultural facilities, contribute to the invasion of these invasive ants and determine their domestic establishment, allowing them to survive in harsh climates^20^.As a result of adversarial impacts and the increasing risk of invasion in South Korea, the RIFA is one of the major concerns of the Animal and Plant Quarantine Agency of Korea, among new threats from invasive species.

Recently, scientists using ‘omic’ technologies have determined which pathways are important for allowing a species of beetle to cope with temperature stress^22^. Transcriptomics and the fast development of novel high-throughput sequencing technologies, such as RNA-Seq, have provided an opportunity to investigate signaling-associated genes and trigger putative function(s) and pathway(s) at low and high temperature stress conditions, in insects^23,24^.

RNA-seq technologies in a New Zealand alpine stick insect demonstrated upregulation of cuticle genes following cuticle modification in response to low temperature was observed^25^. Since 2014, transcriptome analysis using RNA-seq has been used to investigate gene expression changes when coping with thermal stress in several species of insects (*Drosophila virilis*^26^, *Cryptolaemus montrouzieri*^24^, *Microdera punctipennis*^27^, *Nilaparvata lugens, Sogatella furcifera, Laodelphax striatellus*^28^, *Galeruca daurica*^22^, and *Monochamus alternatus*^7^). The findings of such studies demonstrate that cold stress can change the expression levels of hundreds of genes associated with transcription, metabolism, and cuticular organization, especially enzyme-related genes responsible for the upregulation of encoding cytochrome P450s (P450), antioxidative enzymes, and aldehyde dehydrogenase^24,29,30^.

In this study, to produce transcriptomes and analyze changes in transcription regulation associated with cold and heat treatment in *S. invicta*, we used RNA-Seq and de novo transcriptome assemblies. A detailed differential expression analysis identified a variety of candidate genes that could be linked to RIFA's cold and heat tolerance. To verify the RNA-seq results, we used qRT-PCR. We aimed to provide a foundation for the adaptive mechanism as well as a rich resource for finding and identifying new genes involved in the cold and heat stress responses in red imported fire ants.

## Results

### Sequencing, RNA-Seq assembly, and functional annotation

Quality filtering for Illumina raw data (Table S2) was completed to investigate the transcriptome responses to heat and cold stress in *S. invicta*. After transcriptome sequencing of four cDNA samples with Q30 > 94%, 44.53 GB of clean data passed the Illumina consistency filter (Table S3). All high-quality reads (Table S3) were pooled to perform the de novo transcriptome assembly. These contigs were further assembled into 107,264 transcripts with a mean length of 757.72 bp and a N50 of 1504 bp, and 99,085 unigenes with a mean length of 615.38 bp and a N50 of 1051 bp (Tables S4 and S5). The length distribution of unigenes was very similar to the transcript length distribution. This suggests a high-quality assembly, which will serve as a sequence foundation for future research.

### Annotation of predicted proteins

**Figure 1:**
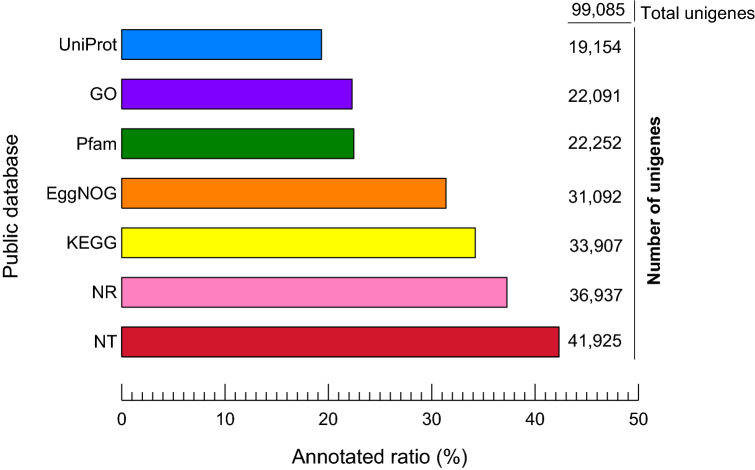
Blasted results for unigenes based on public databases with an *E*-value default cutoff of *E*-value 10^–5^. The X axis shows the ratio (%) of annotated unigenes for each database based on total unigenes. The Y axis shows the number of unigenes that are annotated based on each database.

The assembled unigenes were validated and annotated using BLASTX against five public databases. Genes with a large blast hit to arthropods were detected after annotation. In total, 19,154 unigenes (19.33%) were discovered in at least one public database (UniProt). The NT database had the most matches (41,925 annotated unigenes, 42.31%), followed by the NR database (21,232, 37.28%) (Fig. 1, Table 1). The majority of the unigenes were either unable to be annotated or had uninformative definitions (e.g., putative, unknown, hypothetical, or unnamed protein). According to BLASTX matches in the NR database, the unigene sequences were most similar to gene sequences from *S. invicta* (56.80%), and more than 70% showed similarity with ant genera (*Solenopsis* sp, *Trachymyrmex* sp, *Acromyrmex* sp, Atta sp, *Camponotus* sp, and *Cyphomyrmex* sp). ORFs with a duration of at least 100 amino acids were extracted. At least one ORF was found in 14.86% (14,721) of total expected unigenes (99,085), and 49.3% had a complete open reading frame (Table 2).

**Table 1:** Statistics of annotation analysis of unigenes.

Database	Unigene	300 ≤ length < 1000	Length ≥ 1000
NT_Annotation	41,925	5363	36,441
NR_Annotation	36,937	19,637	6067
Pfam_Annotation	22,252	4461	36
EggNOG_Annotation	31,092	17,091	5823
KO_EUK_Annotation	33,907	19,081	6224
GO_Annotation	22,091	12,752	4536
UniProt_Annotation	19,154	11,116	3858

**Table 2:** Statistics of Open Reading Frame (ORF) prediction.

Assembly	Total unigene	ORF predicted unigene	Single ORF predicted unigene	Multiple ORF predicted unigene	
Merge	99,085	14,721 (14.86%)	13,325 (90.52%)	1396 (9.48%)	

Assembly	# of ORF	Complete	Internal	5ʹ partial	3ʹ partial
Merge	16,235	8004 (49.3%)	3733 (22.99%)	3462 (21.32%)	1036 (6.38%)

### GO and EggNOG analysis for global functional classification

**Figure 2:**
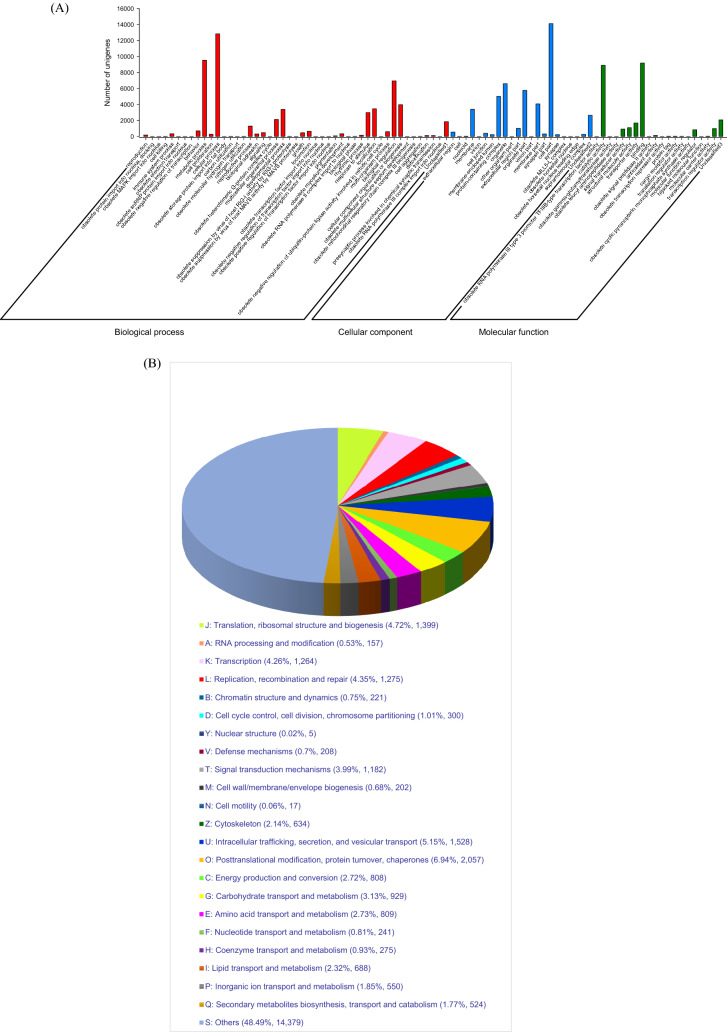
Annotations of the annotated unigenes. (**A**) The Gene Ontology (GO) classification of all annotated unigenes. 22,091 unigenes were assigned to three main GO categories of ‘Biological process with 46 functional subcategories’, ‘Cellular component with 21 functional subcategories’, and ‘Molecular function with 19 functional subcategories. The Y axis shows the number of genes in each category. Some unigenes were not classified and were shown as ‘Unclassified’ in each category. (**B**) The annotated unigenes are mapped to the annotation of the corresponding orthologous groups in the EggNOG (Evolutionary genealogy of genes) database.

The Gene Ontology (GO) database and the EggNOG database were used to identify the annotated unigenes. Since different functional annotations will exist for the same unigene, a total of 22,091 genes have been annotated to GO terms based on BLAST performance. Based on the three main GO groups, ‘biological mechanism’, ‘cell component’, and ‘molecular function’ the GO database yielded 86 GO functional sub-groups. The most frequent GO terms were ‘metabolic process’, ‘cellular process’, ‘cell part’, ‘catalytic activity’ and ‘binding’(Fig. 2A). The most common DEGs coregulated under cold stress according to GO categorization were ‘cellular process’, ‘metabolic process’, ‘biological regulation’, ‘developmental process’, ‘cellular component’, ‘organization or biogenesis’, and localization in biological processes in the biological process category. In the molecular function category, the coregulated DEGs were mostly assigned to ‘cell par’, ‘organelle’, ‘membrane part’ and ‘Protein-containing complex’. For the cellular components category, only ‘binding’ and catalytic activity were significantly enriched (Table 3).

**Table 3:** Significantly enriched GO terms in the DEGs coregulated by cold stresses.

GO term	Unigenes	DEGs (FC > 5)	Corrected *P*-value
**Biological process**			
Cellular process	12,843 (24.1%*)	182 (1.42%**)	7.27E−19
Metabolic process	9546 (17.92%)	133 (1.4%)	0.009931865
Biological regulation	6948 (13.04%)	121 (1.74%)	4.94E−22
Developmental process	3396 (6.37%)	66 (1.94%)	0.00676631
Cellular component organization or biogenesis	3971 (7.45%)	65 (1.63%)	0.006181384
Localization	3458 (6.49%)	52 (1.5%)	0.008235804
Response to stimulus	2982 (5.59%)	39 (1.3%)	1.53E−21
**Cellular component**			
Cell part	14,118 (31.62%)	224 (1.58%**)	0.000326504
Organelle	12,359 (27.85%)	191 (1.54%)	3.46E-20
Membrane part	7688 (17.2%)	145 (1.89%)	0.028381911
Protein-containing complex	6948 (11.21%)	77 (1.1%)	7.65E−27
**Molecular function**			
Binding	9186 (35.38%)	136 (1.48%)	7.53E−20
Catalytic activity	8895 (34.26%)	144 (1.61%)	1.53E−21

* The percent of unigenes in the relevant GO term. ** The percent of DEGs (FC>5) in the relevant GO term.

We discovered functional and biological classification using the EggNOG database. In total, 31,092 unigenes were assigned to 23 EggNOG terms (Fig. 2B) that belonged to three functional classes, including ‘information storage and processing’, ‘cellular processes and signaling’, and ‘metabolism’. The largest number of unigenes were classified as ‘translation, ribosomal structure, and biogenesis’, ‘transcription’, ‘replication, recombination, and repair’, ‘intracellular trafficking, secretion, and vesicular transport’ and ‘post-translational modification, protein turnover, chaperones’ (Fig. 2B).

### Differential gene expression under different temperatures

**Figure 3:**
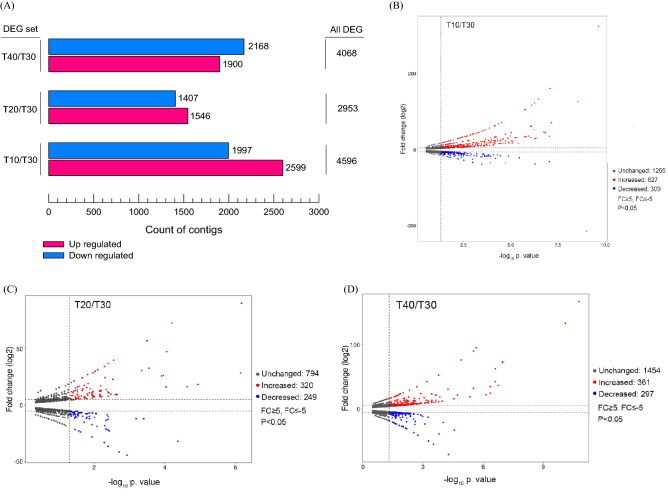
Significant Differentially Expressed Gene (DEG) analysis. (**A**) The number of all up and down-regulated contigs based on p-value < 0.05 and log_2_FC ≥ 2 or log_2_FC ≤ -2 of comparison pairs was plotted. (**B**–**D**) Volcano diagram for distribution of the identified DEGs in three different treatment groups in comparison with T30 as control. Red and blue points represent significant DEGs with *p*-value < 0.05 and log_2_FC ≥ 5 or log_2_FC ≤ -5 and grey ones show those without significant, respectively.

We used the FPKM mapped reads method to measure the expression level of the unigenes. DEGs were found to be up-regulated under different temperature treatments as compared to untreated controls. With a criterion of *p*-value < 0.05 and |log_2_FC| ≥ 2, 4596, 2953, and 4068 unigenes were DEGs for T10, T20, and T40, respectively (Fig. 3A).

A volcano plot with a criterion of *p*-value < 0.05 and log_2_FC ≥ 5 was constructed for each treatment temperature in comparison to the control temperature to identify the most likely temperature-reactive individual genes (Fig. 3B–D). The largest number of expression changes in unigenes were observed in response to T10 (Fig. 3B). GO analysis revealed that most unigenes in the category of ‘biological process’ belong to the ‘cellular process’ sub-group (Table S6), and ‘cellular part’ and ‘binding’ included the largest number of unigenes from the ‘cellular component’ and ‘molecular function’ classifications, respectively (Table S6).

**Figure 4:**
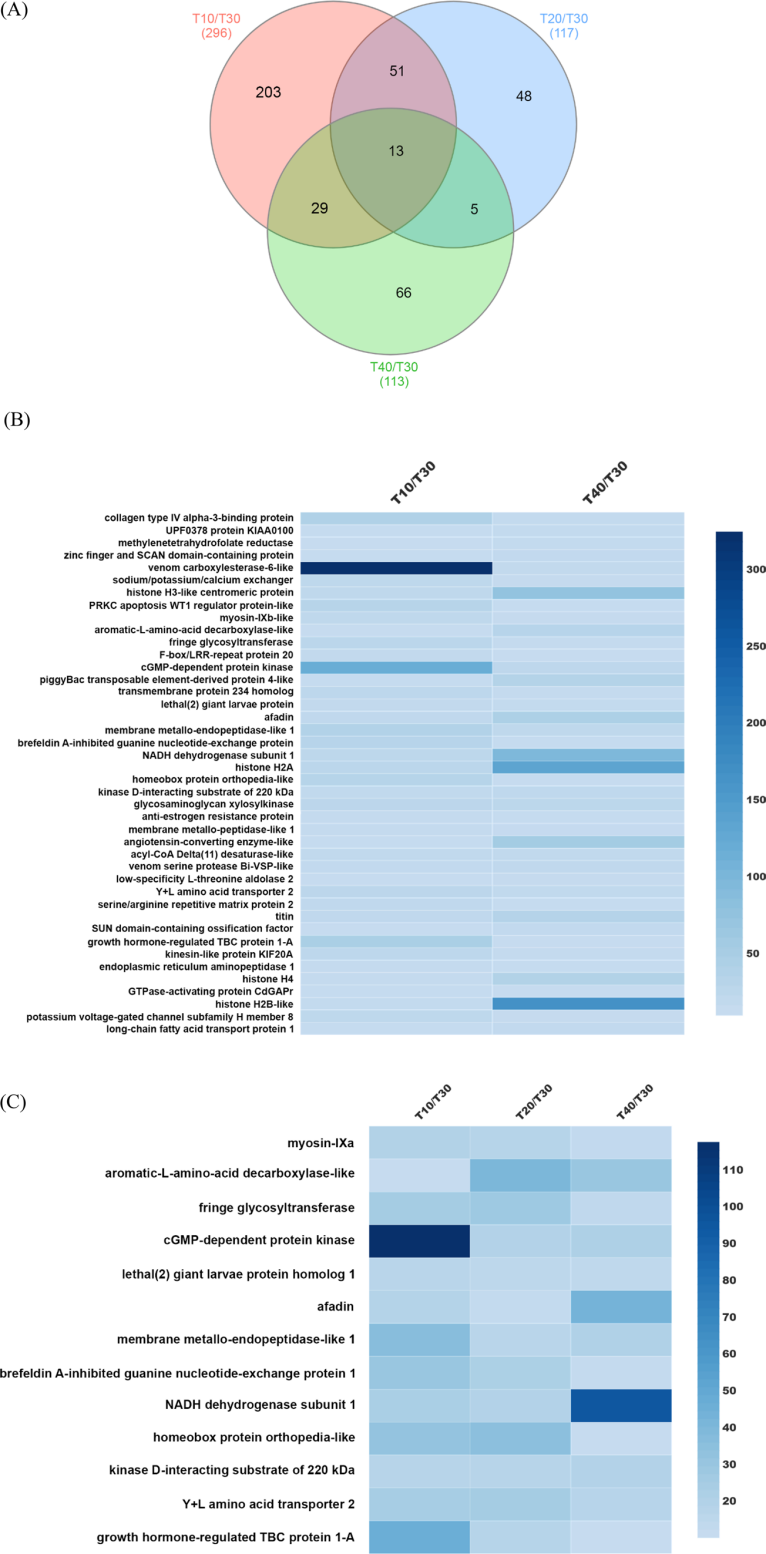
Distribution of the same Differentially Expressed Genes (DEGs) between treatment groups. (**A**) The venn diagram represents the same unigenes (log_2_FC ≥ 10) between all three groups in comparison with T30 as control. (**B**) Heatmap shows expression patterns for same DEGs (p-value < 0.05, log_2_FC ≥ 10) involved in low (10 °C) and high (40 °C) temperature treatment groups in comparison with T30 as control. (**C**) Heatmap represents expression of the same DEGs (p-value < 0.05, log_2_FC ≥ 10) involved in all three groups of temperature treatment groups compared with T30 as control.

To explore the more specific and exclusive genes involved in cold and high temperature stress conditions, a Venn diagram was plotted for T10, T20, and T40 in comparison with T30 as a control group (*p*-value < 0.05). As shown in Fig. 4A, 203 (Table S9), 48 (Table S10), and 66 (Table S11) differentially expressed genes (DEGs) were identified when comparing the control (30 °C) and stressor temperatures of 10, 20, and 40 °C, respectively. There were 13 common DEGs that were consistently up-regulated in all three groups. The same 51 DEGs were up-regulated (FC ≥ 10) between T10 and T20, when compared with T30. There were 29 and 5 DEGs up-regulated (FC ≥ 10) between T10 and T40; T20 and T40, respectively, as compared to the T30 control. We believe two general groups of unigenes are related to temperature fluctuations. The first group includes the unigenes that are presented in all (T10, T20, and T40) or two (T10 and T40) treatment temperatures, and the second group includes the unigenes that are specifically expressed more than 10 times at one temperature (Fig. 4A). To understand the comparative distribution of unigenes in the first group, a heatmap was constructed (Fig. 4B,C). ‘Venom carboxylase-6-like (*p*-value 3.29E−14)’, ‘cGMP-dependent protein kinase (*p*-value 1.02E−09)’ and ‘growth hormone regulated TBC protein 1-A (*p*-value 4.36E−06)’ showed high expression levels when the ants were incubated at 10 °C in comparison with 40 °C and 30 °C controls. Interestingly, ‘histone’ unigenes (histone H3-like centromeric protein (*p*-value 5.71E−06), histone H2A (*p*-value 1.21E−07), histone H4 (*p*-value 1.86E−06), and histone H2B-like (*p*-value 4.2E−05) showed high degrees of fold change at 40 °C in comparison with 10 °C (Fig. 4B). Among 13 matching unigenes, ‘aromatic-l-amino-acid decarboxylase’ and ‘homeobox protein orthopedia-like’ showed lower expression levels at T10 and T40 when compared to those at T20 (Fig. 4C, Table S8).

**Figure 5:**
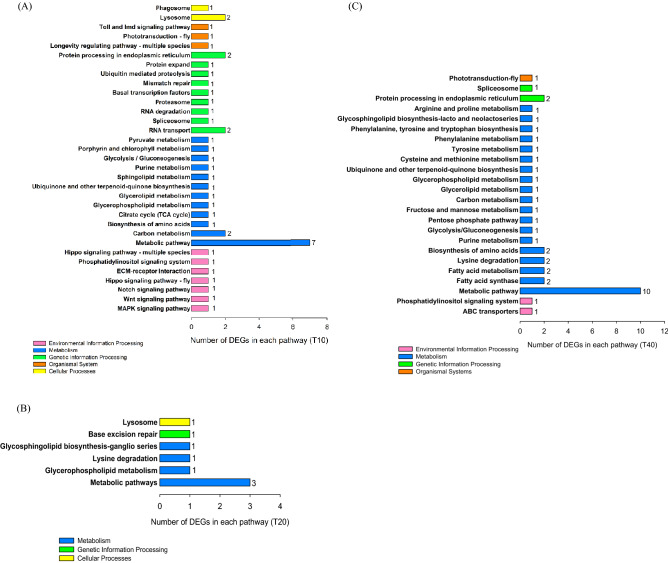
(**A**–**C**) Analysis of significant KEGG pathway enrichment in the specific Differentially Expressed Genes (DEGs) (p-value < 0.05, log_2_FC ≥ 10) co-regulated by temperature stresses including 10, 20, and 40 °C, respectively. The Y axis indicates the KEGG pathways, and the X axis shows the number of specific DEGs for each treatment group in each pathway that is indicated on the right side of the bars.

**Figure 6:**
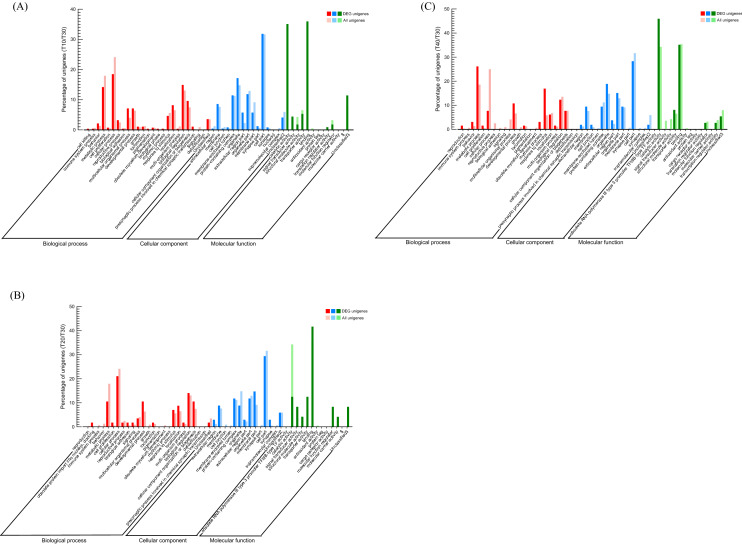
(**A**–**C**) Gene Ontology (GO) annotations of the specific Differentially Expressed Genes (DEGs) (p-value < 0.05, log_2_FC ≥ 10) co-regulated by temperature stresses including 10, 20, and 40 °C, respectively, in comparison to all DEGs. Go terms are summarized into three main categories; ‘Biological process’, ‘Cellular component’, and ‘Molecular function’. The Y axis shows the percentage (%) of unigenes in each category.

Specific unigenes (log_2_FC ≥ 10) for each treatment were the second group of unigenes that were investigated. KEGG and GO analysis were used to validate functional unigenes (Figs. 5 and 6, Tables S8, 9, and 10). KEGG pathway enrichment analysis revealed the primary DEG pathways (Fig. 5A–C). Pathway enrichment was observed among all groups, although the T20 group showed less effects on pathway enrichment than T10 and T40 (Fig. 5B, Table S12). A large number of co-regulated DEGs under cold and high temperature stresses were significantly enriched in the ‘Metabolic pathway’ which in T10 is dominated by ‘pyruvate carboxylase (e-value 0.0)’, ‘diacylglycerol kinase 1 (e-value 0.0)’, ‘hexaprenyldihydroxybenzoate methyltransferase (e-value 0.0)’, ‘sphingomyelin phosphodiesterase 1-like (e-value 2E−180)’, ‘adenylate cyclase type 6 (e-value 8E−171)’, ‘ADP-dependent glucokinase (e-value 0.0)’, and ‘porphobilinogen deaminase-like (e-value 7E−165)’. Another identified enriched-pathway for the T10 group included ‘Lysosome’, which includes ‘CD63 antigen-like (e-value 4E−110)’ and ‘sphingomyelin phosphodiesterase 1-like (e-value 2E−180)’ unigenes. ‘RNA transport pathway’ and ‘carbon metabolism pathway’ are other pathway enriched in the T10 group (Fig. 5A). Three proteins presented in the ‘Metabolic pathway’ in T20 were ‘muscle M-line assembly protein unc-89 (e-value 0.0)’, ‘histone-lysine N-methyltransferase SETMAR-like (e-value 2E−09)’, and ‘acetyl-coenzyme A transporter 1 (e-value 3E−150)’ (Fig. 5B). Additionally, ‘fatty acid synthase (e-value 0.0)’, ‘phosphodiesterase 8A (e-value 0.0)’, ‘procollagen-lysine,2-oxoglutarate 5-dioxygenase 1 (e-value 3E−23)’, ‘fatty acid synthase-like (e-value 1E−22)’, ‘fructose-bisphosphate aldolase-like (e-value 2E−164)’, ‘histone-lysine N-methyltransferase SETMAR-like (3E−95)’, ‘eye-specific diacylglycerol kinase (e-value 0.0)’, ‘tyrosine aminotransferase (e-value 0.0)’, ‘beta-1,3-galactosyltransferase 5 (e-value 0.0)’, and ‘S-adenosylmethionine decarboxylase proenzyme (e-value 2E−92)’ were the transformed components of the metabolic pathway of T40 group. ‘Protein processing in endoplasmic reticulum’, ‘Biosynthesis of amino acids’, ‘Lysine degradation’, ‘Fatty acid metabolism’, and ‘Fatty acid synthase’ are other enriched pathways in the T40 group (Fig. 5C, Table S12).

GO annotation was used to clarify the functions of DEGs that were significantly different (FC ≥ 10) between treatments (Fig. 6). Between T10 and T30 control, the most significant items were ‘Biological Process: multicellular organismal process’, ‘Cellular Component: organelle’ and ‘Molecular Function: catalytic activity and binding’ (Fig. 6A). When comparing T20 and T30 controls, unigenes were assigned to ‘Biological Process: developmental process’, ‘Cellular component: membrane part’ and ‘Molecular Function: binding and molecular function regulator’ (Fig. 6B). The comparison between T40 and T30 control revealed the following significant increase in unigene percentages: ‘Biological Process: response to stimulus’, ‘Cellular component: organelle’ and ‘Molecular Function: catalytic activity’ (Fig. 6C).

### Validation of gene expression profiles by q-PCR

**Figure 7:**
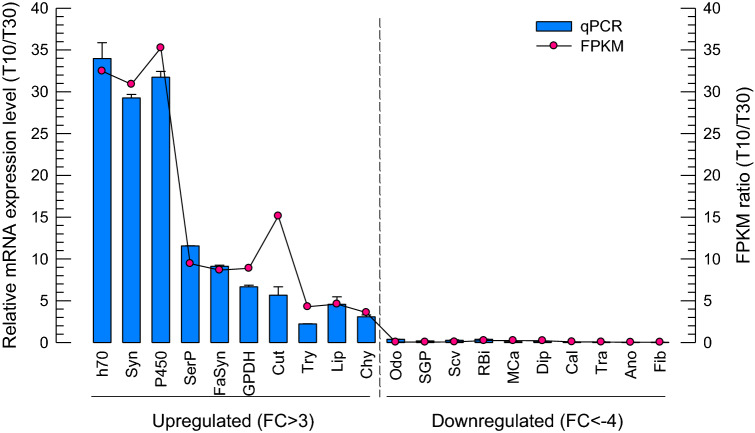
Differentially Expressed Genes (DEGs) validation by qRT-PCR in comparison to corresponding FPKM data detected in RNA-Seq. The relative expression level of each DEG is presented as blue bars and the ratio of − log_2_ FPKM (T10/T30) is plotted as a simple line and red scatters. Relative gene expression (T10/T30) is shown as the ratio of them per *ef1_β.* Each treatment was replicated three times.

qPCR and gel electrophoresis of twenty common DEGs identified in the RNA sequence data were performed to confirm the accuracy and reproducibility of the Illumina RNASeq. The results of qPCR and Illumina FPKM ratio were plotted in Fig. 7. These data demonstrated that expression changes were in the same direction as the qPCR. The Illumina sequencing data was consistent with qPCR data, verifying the reliability and accuracy of the transcriptome analysis. This ensures the RNA-Seq results are considerably reliable for the identification of DEGs under temperature stress, and also the feasibility and sustainability of our further research into these or other DEGs from the transcriptome data.

## Discussion

The red imported fire ant, *Solenopsis invicta*, is one of the most well-known ant invaders. The identification of geographical areas that are climatically vulnerable to *S. invicta* infestation should aid efforts to control the pest ant's spread^16^. *S. invicta* infestation threatens many parts of the world, including broad areas of Europe, Asia, Africa, Australia, and a number of island nations. Officials in charge of quarantine should be on the lookout for any unintentional introductions of this pest into vulnerable areas. As the size of the infestation increases, the cost of eradication rises sharply, and large infestations can be difficult to eradicate^16^. Researchers have long assumed that introduced *S. invicta* populations in the Southeast would not spread to higher latitudes and elevations because their pre-adaptation to the subtropical climate of their native range would render them unable to tolerate prolonged cold weather exposure^31–33^. However, over the last 5 years, at least *S. invicta* colonies have been found in the Blue Ridge Escarpment at altitudes > 1220 m where the temperature in Macon County, North Carolina, reached an abnormal minimum of − 16.3 °C in 2019^34^. With rising elevation, *S. invicta*'s cold and heat tolerance thresholds decreased, suggesting a physiological capacity to withstand colder temperatures. The lipid content of *S. invicta* did not change with elevation, indicating that cold acclimation had no metabolic effects on the ants. These findings indicate that *S. invicta* is not as resistant to cold temperatures as previously believed, and that it will continue to adapt and spread to higher elevations and latitudes^35^. Comprehensive investigation of gene expression regulation under temperature stress is very important to understand the biochemical and physiological adaptation processes of invasive insect pests^22^. In this study, a comprehensive transcriptome analysis and characterization of the gene expression profiles of *S. invicta* under cold and high temperature stress were evaluated. Through the analysis of DEGs, transcriptome changes in *S. invicta* adult ants were revealed. Using RNA-seq techniques, four transcriptomes were de novo assembled from the adult stages of RIFA which were exposed to four different temperatures (10, 20, 30, and 40 °C), and 19,154 unigenes (19.33%) were successfully annotated from at least one public database (UniProt) (Table 1). The results are in line with other transcriptome projects using Illumina technology^36–38^. More than 70% similarity with the ant genus was found and 56.80% of the unigene sequences were most similar to gene sequences from *Solenopsis invicta*. In this study, DEGs from adult RIFA subjected to various treatment temperatures (10, 20, and 40 °C) were compared to a 30 °C control group in this analysis. The majority of DEGs were observed at T10, followed by T40, both of which expressed a greater DEG distribution than T20 (Fig. 3A). As mentioned earlier, this is consistent with proteomics data from *Locusta migratoria* under high and low temperature stress^39^. To identify specific genes associated with response to temperature, the number of unigenes with log_2_FC ≥ 10 was clarified by the Venn diagram, and KEGG analysis was conducted to determine the probability of function in pathway enrichment. KEGG analysis revealed that of 203 specific cold-regulated DEGs (associated with T10), 41 DEGs were enriched in the KEGG pathways; ‘Metabolic pathway’, ‘Carbon metabolism’, ‘Citrate cycle (TCA)’, ‘RNA transport’, and ‘Lysosome’. Interestingly, in T20 and T40, the ‘Metabolic pathway’ included more DEGs than other pathways (Fig. 5). ‘Purine metabolism’, ‘Spliceosome’, ‘Lysosome’, ‘RNA degradation’, ‘Glycolysis/Gluconeogenesis’, ‘Pyruvate metabolism’, ‘Phagosome’, ‘Sphingolipid metabolism’, ‘RNA transport’, ‘Glycerolipid metabolism’, ‘Carbon metabolism’, ‘ECM-receptor interaction’ are pathways that demonstrate similar results as those of investigations on transcriptome responses to cold stress in the carpenter moth, *Eogystia hippophaecolus*^36^, and the a chrysomelid beetle, *Galeruca daurica*^22^. The transcriptome analysis revealed that the expression of ‘Glycolysis’ and ‘TCA cycle pathways’ are up-regulated in a similar manner to the braconid wasp, *Aphidius colemani,* when exposed to low temperatures^40^. When RIFA was exposed to the highest temperature (40 °C), ‘Tyrosine metabolism’, ‘Phenylalanine metabolism’, ‘Cysteine and Methionine metabolism’, ‘Spliceosome’, ‘Protein processing in endoplasmic reticulum’, and ‘Metabolic pathway’, were found to be enriched pathways that are similarly enriched in three species’ of rice plant hopper when exposed to 37 °C^28^. ‘Fatty acid synthase’ and ‘Fatty acid metabolism’, are two of the main pathways of RIFA when exposed to high temperatures. The expression of fatty acids as hydrophobic agents allows insects to avoid water loss in warmer regions of the globe^41^. *Gomphocerus sibiricus* is known to increase its levels of oleic acid, linoleic acid, and glycerin in response to high temperatures, a process that may reduce mortality due to excessive evaporation of body moisture^42^. In our study on RIFA, ‘Amino acid metabolism’ was clearly up-regulated during high-temperature stress. It is suggested that amino acid metabolism provides heat resistance in RIFA similar to that of results that have been reported for *Locusta migratoria*^39^ when exposed at 40 °C. Due to the synthesis of immune proteins and defense enzymes, insects seek out and consume numerous free amino acids when coping with stress conditions such as high temperature, low temperature, and fungal invasion^43^. The synthesis and metabolism of amino acids are necessary to produce a significant number of amino acids, which make available the raw materials necessary for the synthesis of heat-resistant proteins^39^.

In this study, two cuticular protein unigenes were identified from 203 co-regulated DEGs under cold temperature stress (Table S9). Cuticular protein gene expression has been observed in studies of other insects such as beetles, moths, planthoppers, and stick insects when exposed to cold temperature stress^22,28,36,44^. Although the physiological role of cuticular proteins in insect cold hardiness has not yet been identified, it seems insect cuticle may play an important role in insects when coping with low temperatures^22,25,28,45,46^.

According to GO analysis (Fig. 6A), ‘Antioxidant activity’ was enriched at low temperatures suggest that it might contribute to RIFA ability to resist oxidative stress damage at low temperatures^22^, or their potential for cell preservation via antioxidant defense when challenged by environmental complexity^47^. In addition, one cytochrome P450 was identified that is up-regulated exclusively under low temperatures (Table S9). Meanwhile, NADH dehydrogenase subunit 1 was up-regulated at all treated temperatures (Fig. 5C, Table S8). These two proteins are the main enzymes in the antioxidant activity pathway^36^. In a comparative analysis of the transcriptional responses to low and high temperatures in three rice planthopper species, some cytochrome P450 genes were up-regulated under both low and high temperatures, which suggests cold and heat stress increase oxidative stress in the insect body^28^.

Heat shock proteins (HSPs) are another important protein that insects use as critical physiological products when under abiotic stress conditions^39^. From earlier studies, it was believed that *Hsp* is associated with biological cold and heat resistance^48,49^. HSPs are molecular chaperones, that play important physiological roles, including correct folding of proteins, prevention of protein denaturation, and degradation of misfolded or condensed proteins, and maintenance of correct protein conformation^50,51^. In this study, we identified one specific *Hsp70* that up-regulated about a 12-fold change when RIFA was exposed at 10 °C (Table S9). Two pathways, including ‘Protein export’ and ‘Protein processing in endoplasmic reticulum’, were enriched under *Hsp70* gene expression at low temperatures (Fig. 5A, Table S12). Interestingly, heat shock protein 83 was found only in the 40 °C treatment group that was up-regulated about 11 times (Table S11). Many studies have confirmed that the expression of *Hsp* genes can be up-regulated by cold and heat stimulus^50,52^. To assist the resistance to temperature stress, the *Hsp60* gene expression in *Stegobium paniceum* significantly increases under high-and low-temperature stress^53^. Three *Hsp90* and four *Hsp70* were up-regulated by cold stress and were differentially expressed in the desert beetle, *Microdera punctipennis*^27^. The differences in *Hsp*, insect species, sex of organism, and intensity of temperature are important factors related to *Hsp* expression level in insects^22,54^.

In conclusion, we compared the transcriptomes of *S. invicta* under high-and low-temperature stresses using RNA-Seq technology based on high-throughput sequencing. Comparative transcriptome analysis identified many genes, and a large number of changes were discovered in metabolic pathways through GO and KEGG enrichment analysis. Our data will facilitate further molecular investigations and genomic research. Many novel relationships between high-and low-temperature and significantly up-regulated genes were identified in this study (Tables S7–11). These newly found genes may be important for RIFA overwintering and adaptation potential in new environments as well as quarantine areas.

## Materials and methods

### Insect rearing, exposure temperatures and sample preparation

*Solenopsis invicta* colonies were collected in Somerville, TX, US (30°31′13″ N, 96°25′33″W) and imported to Korea according to the Plant Protection Act. All insects were reared at the Plant Quarantine Technology Center laboratories (Animal and Plant Quarantine Agency, Gimcheon, Korea). To prevent insects escape, all laboratories are equipped with an automatic wind curtain and sticky floor mats at entrance. Ant colonies were maintained at 25 ± 1 °C. Plastic trays (25 (H) × 30 × 35 cm^3^) containing each test colony were placed in larger holding trays (35 (H) × 45 × 65 cm^3^). Talcum powder was dusted on the top 10 cm interior edge of the trays and along the bottom of the larger holding trays so that any escaping ants would be trapped inside the holding trays. Ants were fed a 20% sucrose solution, mealworms (*Tenebrio molitor* larvae), and an artificial diet described by Dussutour and Simpson^55^. Water was provided ad libitum. Colonies contained dealated mated queens, alate queens, males, brood (eggs, larvae, and pupae) and workers. To perform transcriptomic analysis, medium sized workers were incubated at 10, 20, and 40 °C for 24 h. Ants incubated at 30 °C were considered as the control group. A temperature recorder (Lutron, BTM-4208SD) was used to check the output temperature, which was recorded every 2 s for 24 h. According to the collected data, the incubator's variance is 0.5°. Each treatment was replicated three times. After the temperature treatment, ten ants from each group were immediately frozen in liquid nitrogen and stored at − 80 °C for subsequent experiments.

### RNA extraction and RT-qPCR

RNA samples were extracted from the whole body of *S. invicta* adult worker ants using Trizol reagent (Invitrogen, Carlsbad, CA, USA) according to the manufacturer's instructions. After RNA extraction, it was resuspended in nuclease-free water and quantified using a spectrophotometer (NanoDrop, Thermo Scientific, Wilmington, DE, USA). cDNA was then synthesized from RNA (1 μg) using RT PreMix (Intron Biotechnology, Seoul, Korea) containing an *oligo dT* primer according to the manufacturer's instructions. All quantitative PCRs (qPCRs) in this study were determined using a real-time PCR machine (CFX Connect Real-Time PCR Detection System, Bio-Rad, Hercules, CA, USA) and iQ SYBR Green Supermix (Bio-Rad, Hercules, CA, USA) according to the guidelines provided by the manufacturer. The reaction mixture (20 μL) contained 10 μL of iQ SYBR Green Supermix, 1 μL of cDNA template (100 ng), 1 μL each of forward and reverse prim (Table S13), and 7 μL nuclease free water. RT-qPCR cycling began with a 95 °C heat treatment for 10 min followed by 40 cycles of denaturation at 94 °C for 30 s, annealing at 52 °C for 30 s, and extension at 72 °C for 20 s. The expression level of *Ef1_β* as a reference gene was used to normalize target gene expression levels^56^ under different treatments. PCR products were assessed by melting curve analysis. Quantitative analysis was performed using comparative CT (2^−ΔΔCT^) method^57^.

### Illumina sequencing

To obtain short-read RNA sequences, Illumina sequencing was performed at Macrogen (Seoul, Korea). Each library was constructed from 1 μg total RNA from the whole body of 5 individuals (not pooled) of *S. invicta* adults per treatment using the TruSeq Stranded mRNA LT Sample Prep Kit (Illumina, San Diego, USA) and sequenced using the HiSeq 4000 System (Illumina, San Diego, USA) with a 101 bp pair end read (Table S1).

### *De novo* assembly

Illumina short reads were quality-filtered and adapter-trimmed using Trimmomatic v0.38 (http://www.usadellab.org/cms/?page=trimmomatic). FastQC v0.11.7 (http://www.bioinformatics.babraham.ac.uk/projects/fastqc/) was used to check data quality before and after trimming. After the removal of low-quality reads, an Illumina-based de novo transcriptome assembly was performed using Trinity version trinity rnaseq r20140717, bowtie 1.1.2^58^. Trimmed reads for every sample were merged into one file to construct a combined reference. The de novo assembly of merged data was carried out using Trinity with default parameters and assembled into transcript contigs^59^. The total number of genes, transcripts, GC content, max/min/median/average contig length, and total assembled bases were summarized. Trinity groups transcripts into clusters based on shared sequence content. For assembled genes, the longest contigs of the assembled contigs are filtered and clustered into non-redundant transcripts using CD-HIT version 4.6 (http://weizhongli-lab.org/cd-hit)^60^. These transcripts were defined as ‘unigenes’ which are used for predicting ORFs (Open Reading Frames), annotating against several known sequence databases, and analyzing differentially expressed genes (DEGs). The ORF prediction for unigenes was performed using TransDecoder version 3.0.1 (https://github.com/TransDecoder/TransDecoder/wiki)^61^ to identify candidate coding regions within transcript sequences. After extracting ORFs that were at least 100 amino acids long, the TransDecoder predicted the likely coding regions. Trimmed reads for each sample were aligned to the assembled reference using the Bowtie program. For the differentially expressed gene analysis, the abundances of unigenes across samples were estimated into read count as an expression measure by the RSEM algorithm (RSEM version v1.2.29, bowtie 1.1.2, http://deweylab.github.io/RSEM/, (Li and Dewey 2011)^62^).

### Gene functional annotation

For functional annotation, unigenes were searched against Kyoto Encyclopedia of Genes and Genomes (KEGG) v20190104 (http://www.genome.jp/kegg/ko.html)^63^, NCBI Nucleotide (NT) v20180116 (https://www.ncbi.nlm.nih.gov/nucleotide/)^22^, Pfam v20160316 (https://pfam.xfam.org/)^64^, Gene ontology (GO) v20180319 (http://www.geneontology.org/)^65^, NCBI non-redundant Protein (NR) v20180503 (https://www.ncbi.nlm.nih.gov/protein/)^66^, UniProt v20180116 (http://www.uniprot.org/)^67^ and EggNOG (http://eggnogdb.embl.de/)^68^ using BLASTN of NCBI BLAST and BLASTX of DIAMOND version 0.9.21 (https://github.com/bbuchfink/diamond) with an *E*-value default cutoff of 10^–5^.

### Differential gene expression analysis

A quality check was conducted for all samples, so that if more than one read count value was zero, it was not included in the analysis. Gene expression levels were measured in the RNA-Seq analysis as fragments per kilobase of transcript per million mapped reads (FPKM)^69^. Multiple testing was corrected for in all statistical tests using the Benjamini–Hochberg false discovery rate with the following parameter values: FDR < 0.01^36^. In order to reduce systematic bias, we estimated the size factors from the count data and applied Relative Log Expression (RLE) normalization with the DESeq_2_ R library. Using each sample’s normalized value, the high expression similarities were grouped together by Hierarchical Clustering Analysis and graphically shown in a 2D plot to show the variability of the total data using Multidimensional Scaling Analysis. Significant unigene results were analyzed as Up and Down-regulated count by log_2_FC ≥ 5,  ≤ − 5 and ≥ 10, distribution of expression levels between the two groups was plotted as Volcano plot (https://huygens.science.uva.nl/VolcaNoseR)^70^ and simple bar plots. The DEGs were then used for GO and KEGG enrichment analysis using the edgeR exact test. The software topGO was used to carry out GO enrichment analysis. All DEGs were aligned to terms in the KEGG database and searched for significantly enriched KEGG terms. Heat maps were generated using the online tool Heatmapper (http://www.heatmapper.ca/expression/)^71^.

### Quantitative RT-PCR validation

The twenty genes in response to cold treatment (T10) were chosen for validation using qRT-PCR. Ten up-regulated DEGs were included; hsp70 like-protein (h70; c412839_g5_i2), synapsin (Syn; c408336_g1_i2), cytochrome P450 (P450; c407395_g2_i1), serine protease (SerP; c391510_g1_i1), fatty acid synthase like (FaSyn; c412971_g1_i1), glycerol-3-phosphate dehydrogenase (GPDH; c391490_g1_i2), cuticle protein (Cut; c385485_g1_i2), trypsin-2-like (Try; c395664_g1_i3), lipase-3-like (Lip; c412461_g4_i1), and chymotrypsin (Chy; c414255_g1_i1). Ten DEGs that showed down-regulation at T10 in comparison to T30 controls were validated by qPCR, including; general odorant-binding protein 72 (Odo; c410048_g3_i1), small G protein signaling modulator 3 homolog (SGP; c422133_g1_i1), scavenger (Scv; c412512_g2_i1), RNA binding protein 33 like (RBi; c375277_g1_i1), monocarboxylate transporter 1-like (MCa; c411974_g1_i2), dipeptidase 1-like (Dip; c412614_g1_i6), calmodulin-like protein 4 (Cal; c400490_g2_i1), transmembrane channel-like protein 2 (Tra; c412553_g2_i3), anoctamin-4 (Ano; c409055_g3_i1), and fibrinogen silencer-binding protein like (Fib; c411675_g1_i1). To do that, *S. invicta* adults were incubated at 10° and 30 °C for 24 h in two separate groups that included 10 ants. RNA extraction and cDNA synthesis were performed according to the “RNA extraction and RT-qPCR” section. Specific primers were designed using the Primer Quest tool (http://www.idtdna.com) (Table S13). The expression level of *Ef1_β* was used as a reference gene and to normalize target gene expression levels under different treatments^56^. PCR products were assessed by melting curve analysis. Quantitative analysis was performed using the comparative CT (2^−ΔΔCT^) method^57^. Finally, the data was compared according to the ratio of FPKM and the ratio of mRNA expression levels for all selected genes.

## Supplementary Information

Supplementary Information.
