# Supplementary material for: Comparison of gene expression in the red imported fire ant (Solenopsis invicta) under different temperature conditions
Source: Sci Rep. 2021 Aug 13;11:16476. doi: 10.1038/s41598-021-95779-w (PMC8363622; doi:10.1038/s41598-021-95779-w)
Supplement: Supplementary file 1 — Supplementary Information. [file 41598_2021_95779_MOESM1_ESM.docx]

***Scientific Reports***

**Comparison of gene expression in the red imported fire ant (*Solenopsis invicta*) under different temperature conditions**

**Mohammad Vatanparast^1^, Robert T. Puckett^2^, Deuk-Soo Choi^1^, and Youngjin Park^1,*^**

*^1^Plant Quarantine Technology Center, Animal and Plant Quarantine Agency, Gimcheon 39660, Korea*

*^2^Department of Entomology, Texas A&M University, College Station, TX 77843, USA*

^*^Corresponding author

Email) [parky1127@korea.kr](mailto:parky1127@korea.kr)

**Supplementary table legends**

**Table S1.** Project information of transcriptome analysis.

**Table S2**. Summary of data production. Raw data statistics results. Q20 and Q30 are ratio (%) of bases that have phred quality score greater than or equal to 20 or 30, respectively.

**Table S3.** Summary of data production. Trimming data statistics results. Adapter sequences were removed. Q20 and Q30 are ratio (%) of bases that have phred quality score greater than or equal to 20 or 30, respectively.

**Table S4.** *De novo* transcriptome assembly statistics. The *de novo* assembly of merged data was carried out using Trinity software. N[x] length statistic: At least x% of the assembled transcript nucleotides are found in contigs that are at least of Nx length.

**Table S5.** Clustering transcripts into unigenes. Longest contigs of the assembled contigs are filtered and clustered into the non-redundant transcripts using CD-HIT-EST program. N[x] length statistics: At least x% of the assembled transcript nucleotides are found in contigs that are at least of Nx length.

**Table S6.** Significantly enriched GO terms in the DEGs co-regulated by cold stresses, 10℃, (T10) in comparison with T30.

**Table S7.** Same DEGs between T10 and T40 in comparison with T30 that up-regulated (log_2_FC ≥ 10).

**Table S8.** Same DEGs between T10, T20 and T40 in comparison with T30 that up-regulated (log_2_FC ≥ 10).

**Table S9.** Specific and core T10 associated DEGs in comparison with T30 that up-regulated (log_2_FC ≥ 10).

**Table S10.** Specific and core T20 associated DEGs in comparison with T30 that up-regulated (log_2_FC ≥ 10).

**Table S11.** Specific and core T40 associated DEGs in comparison with T30 that up-regulated (log_2_FC ≥ 10).

**Table S12.** Associated genes related to pathways enrichment base on KEGG classification (log_2_FC ≥ 10).

**Table S13.** Primer sequences used in this study.

Table S1.

| **Project information** | |
| --- | --- |
| Read Length | 101 |
| Library Kit | TruSeq Stranded mRNA LT Sample Prep Kit |
| Library Protocol | TruSeq Stranded mRNA Sample Preparation Guide, Part # 15031047 Rev. |
| Reagent | TruSeq 3000 4000 SBS Kit v3 |
| Sequencing Protocol | HiSeq 3000 4000 System User Guide Document # 15066496 v05 HCS 3.3.52 |
| Sequencing Control Software | HCS 3.3.52 |

Table S2.

| Index | Sample ID | Total read bases | Total reads | Throughput  (Gb) | GC (%) | Q20 (%) | Q30 (%) |
| --- | --- | --- | --- | --- | --- | --- | --- |
| 1 | T10 | 11,213,312,698 | 111,022,898 | 11.2 | 37.04 | 97.84 | 94.42 |
| 2 | T20 | 11,084,552,242 | 109,748,042 | 11.08 | 33.48 | 98.13 | 95.09 |
| 3 | T30 | 11,971,722,506 | 118,531,906 | 11.9 | 35.26 | 98.13 | 95.05 |
| 4 | T40 | 10,376,710,912 | 102,739,712 | 10.35 | 36.93 | 98.39 | 95.55 |

Table S3.

| Index | Sample ID | Total read bases | Total reads | Throughput  (Gb) | GC (%) | Q20 (%) | Q30 (%) |
| --- | --- | --- | --- | --- | --- | --- | --- |
| 1 | T10 | 10,789,615,881 | 108,337,848 | 10.8 | 37.09 | 98.64 | 95.77 |
| 2 | T20 | 10,712,427,401 | 107,793,124 | 10.75 | 33.53 | 98.85 | 96.29 |
| 3 | T30 | 11,544,203,295 | 115,632,192 | 11.55 | 35.27 | 98.84 | 96.29 |
| 4 | T40 | 10,081,809,175 | 100,840,410 | 10.1 | 36.95 | 98.95 | 96.52 |

Table S4.

| Assembly | Merge | |
| --- | --- | --- |
|  | All transcript  contigs | Only longest  isoform per'gene' |
| Total trinity 'genes' | 107,264 | 107,264 |
| Total trinity transcripts | 138,716 | 107,264 |
| Percent GC | 39.21 | 38.92 |
| N90 | 267 | 238 |
| N80 | 409 | 302 |
| N70 | 668 | 416 |
| N60 | 1,056 | 614 |
| N50 | 1,504 | 965 |
| N40 | 1,994 | 1,495 |
| N30 | 2,543 | 2,149 |
| N20 | 3,274 | 2,975 |
| N10 | 4,405 | 4,247 |
| Maximum contig length | 14,843 | 14,843 |
| Minimum contig length | 201 | 201 |
| Median contig length | 352.0 | 303.0 |
| Average contig length | 757.72 | 595.59 |
| Total assembled bases | 105,107,920 | 63,885,511 |

Table S5.

| Assembly | Merge | |
| --- | --- | --- |
|  | Only longest isoform  per'gene' | Clustered Contig  ‘Unigene’ |
| Total 'genes' | 107,264 | 99,085 |
| Percent GC | 38.92 | 39.03 |
| N90 | 238 | 240 |
| N80 | 302 | 311 |
| N70 | 416 | 440 |
| N60 | 614 | 664 |
| N50 | 965 | 1,051 |
| N40 | 1,495 | 1,597 |
| N30 | 2,149 | 2,251 |
| N20 | 2,975 | 3,070 |
| N10 | 4,247 | 4,319 |
| Maximum contig length | 14,843 | 14,843 |
| Minimum contig length | 201 | 201 |
| Median contig length | 303.0 | 306.0 |
| Average contig length | 595.59 | 615.38 |
| Total assembled bases | 63,885,511 | 60,974,807 |

Table S6.

| GO term | Unigenes | DEGs (FC > 5) | Corrected  *P*-value |
| --- | --- | --- | --- |
| **Biological process** |  |  |  |
| Cellular process | 12,843 (24.1%*) | 182 (1.42%**) | 7.27E-19 |
| Metabolic process | 9,546 (17.92%) | 133 (1.4%) | 0.009931865 |
| Biological regulation | 6,948 (13.04%) | 121 (1.74%) | 4.94E-22 |
| Developmental process | 3,396 (6.37%) | 66 (1.94%) | 0.00676631 |
| Cellular component organization or biogenesis | 3971 (7.45%) | 65 (1.63%) | 0.006181384 |
| Localization | 3458 (6.49%) | 52 (1.5%) | 0.008235804 |
| Response to stimulus | 2982 (5.59%) | 39 (1.3%) | 1.53E-21 |
| Multicellular organismal process | 2125 (3.98%) | 38 (1.78%) | 8.35E-14 |
| Reproductive process | 1299 (2.43%) | 32 (2.46%) | 3.57E-20 |
| Behavior | 690 (1.29%) | 20 (2.89%) | 8.46E-20 |
| Unclassified | 1851 (3.47%) | 18 (0.97%) | 2E-20 |
| Locomotion | 651 (1.2%) | 16 (2.45%) | 1.79E-17 |
| Growth | 457 (0.85%) | 13 (2.84%) | 1.91E-18 |
| Cell proliferation | 270 (0.5%) | 10 (3.7%) | 3.14E-14 |
| Biological adhesion | 308 (0.58%) | 9 (2.92%) | 9.61E-19 |
| Signaling | 478 (0.89%) | 9 (1.88%) | 7.67E-18 |
| Immune system process | 336 (0.63%) | 5 (1.49%) | 2.65E-16 |
| Multi-organism process | 601 (1.12%) | 5 (0.83%) | 1.3E-12 |
| Presynaptic process, synaptic transmission | 114 (0.2 %) | 4 (3.5%) | 1.24E-11 |
| Obsolete mycelium development | 329 (0.61%) | 4 (1.21%) | 7.56E-15 |
| Rhythmic process | 143 (0.27%) | 3 (2.1%) | 2.25E-11 |
| Pigmentation | 72 (0.13 %) | 2 (2.7%) | 3.16E-13 |
| Biological phase | 15 (0.028%) | 1 (6.6%) | 2.78E-15 |
| Cell killing | 11 (0.02%) | 1(9.1%) | 1.22E-14 |
| Reproduction | 174 (0.32%) | 1 (0.57%) | 2.74E-13 |
|  |  |  |  |
| **Cellular component** |  |  |  |
| Cell part | 14,118 (31.62%^*^) | 224 (1.58%^**^) | 0.000326504 |
| Organelle | 12,359 (27.85%) | 191 (1.54%) | 3.46E-20 |
| Membrane part | 7,688 (17.2%) | 145 (1.89%) | 0.028381911 |
| Protein-containing complex | 6,948 (11.21%) | 77 (1.1%) | 7.65E-27 |
| Unclassified | 2,648 (5.93%) | 20 (0.75%) | 1.15E-12 |
| Synapse | 527 (1.18%) | 17 (3.2%) | 6.54E-12 |
| Extracellular region | 1549 (3.47%) | 16 (1.03%) | 7.15E-12 |
| Cell junction | 398 (0.89%) | 5 (1.25%) | 2.88E-12 |
| Supramolecular complex | 264 (0.59%) | 5 (1.89%) | 1.65E-09 |
|  |  |  |  |
| **Molecular function** |  |  |  |
| Binding | 9,186 (35.38%) | 136 (1.48%) | 7.53E-20 |
| Catalytic activity | 8,895 (34.26%) | 144 (1.61%) | 1.53E-21 |
| Unclassified | 2,071 (7.97%) | 20 (0.96) | 5.66E-12 |
| Transporter activity | 1,690 (6.5 %) | 22 (1.3%) | 6.64E-12 |
| Structural molecule activity | 1,102 (4.24%) | 11 (0.99%) | 9.25E-12 |
| Signal transducer activity | 927 (3.57%) | 18 (1.94%) | 8.87E-12 |
| Molecular function regulator | 832 (3.2%) | 9 (1.08%) | 2.39E-11 |
| Transcription regulator activity | 989 (3.80%) | 6 (0.60%) | 1.75E-10 |
| Translation regulator activity | 24 (0.09%) | 1 (4.16%) | 6.84E-10 |

Table S7.

| **No** | **Contig** | **Gene** | **Gene bank accession number** | **Target length** | **Identites (%)** | **e-value** | **Corrected *P*-value** | | **Log_2_FC** | | **FPKM** | |
| --- | --- | --- | --- | --- | --- | --- | --- | --- | --- | --- | --- | --- |
|  |  |  |  |  |  |  | T10/T30 | T40/T30 | T10/T30 | T40/T30 | T10/T30 | T40/T30 |
| 1 | c99256_g1_i1 | collagen type IV alpha-3-binding protein | XM_011170728 | 3625 | 99 | 0 | 1.57E-05 | 0.004567 | 39.26 | 13.53 | 40.33 | 19.67 |
| 2 | c456300_g1_i1 | UPF0378 protein KIAA0100 | XM_011173917 | 6643 | 100 | 3E-56 | 0.025273 | 0.004567 | 11.31 | 13.53 | 12.62 | 22 |
| 3 | c455340_g1_i1 | methylenetetrahydrofolate reductase | XM_011070059 | 1305 | 83 | 0.000002 | 0.006189 | 0.000341 | 10.191 | 13.80 | 10.86 | 22.12 |
| 4 | c446832_g1_i1 | zinc finger and SCAN domain-containing protein | XM_012022284 | 8236 | 89 | 9E-147 | 0.025273 | 0.002454 | 11.31 | 15.10 | 12.7 | 24.6 |
| 5 | c440305_g1_i1 | venom carboxylesterase-6-like | XM_011173516 | 1964 | 100 | 5E-44 | 3.29E-14 | 0.00182 | 324.4 | 15.9 | 358.23 | 25.38 |
| 6 | c440174_g1_i1 | sodium/potassium/calcium exchanger | XM_011171793 | 4616 | 99 | 0 | 0.00136 | 0.008799 | 20.25 | 11.95 | 22.3 | 19 |
| 7 | c439941_g1_i1 | histone H3-like centromeric protein | XM_011167451 | 490 | 100 | 0 | 5.71E-06 | 1.84E-11 | 19.53 | 73.33 | 19 | 103.2 |
| 8 | c432043_g1_i1 | PRKC apoptosis WT1 regulator protein-like | XM_011168341 | 2851 | 99 | 4E-76 | 0.000105 | 0.012384 | 30.31 | 11.16 | 32.89 | 17.33 |
| 9 | c416098_g1_i1 | myosin-IXa | XM_011166752 | 3540 | 100 | 2E-83 | 0.00182 | 0.008799 | 19.13 | 11.95 | 21.21 | 19.29 |
| 10 | c413076_g2_i2 | aromatic-L-amino-acid decarboxylase-like | XM_011163525 | 2087 | 98 | 1E-99 | 0.036674 | 3.51E-05 | 10.19 | 29.32 | 11.11 | 47.5 |
| 11 | c412792_g2_i1 | fringe glycosyltransferase | XM_011169756 | 2273 | 100 | 0 | 0.000455 | 0.006311 | 24.72 | 12.74 | 29 | 21 |
| 12 | c411681_g1_i1 | F-box/LRR-repeat protein 20 | XM_011165727 | 4007 | 100 | 1E-112 | 0.004567 | 0.017602 | 15.78 | 10.37 | 17.96 | 18.24 |
| 13 | c411453_g2_i1 | cGMP-dependent protein kinase | XM_011160965 | 3184 | 100 | 7E-80 | 1.02E-09 | 0.000351 | 117.54 | 20.63 | 122.17 | 30.67 |
| 14 | c411119_g1_i1 | piggyBac transposable element-derived protein 4-like | soc:105194817 | 83 | 92 | 6E-18 | 0.003627 | 2.86E-07 | 11.38 | 34.22 | 12.147 | 53 |
| 15 | c410652_g1_i1 | transmembrane protein 234 homolog | XM_011177131 | 1005 | 99 | 6E-131 | 4.8E-05 | 0.000528 | 22.11 | 12.97 | 24.227 | 20.44 |
| 16 | c410115_g1_i1 | Lethal (2) giant larvae protein | XM_011170098 | 4470 | 100 | 1E-62 | 0.004567 | 0.004567 | 15.78 | 13.53 | 18.67 | 23 |
| 17 | c406492_g2_i3 | afadin | XM_012677511 | 9826 | 78 | 0 | 0.002454 | 1.9E-06 | 18.01 | 42.74 | 22.5 | 75.5 |
| 18 | c405854_g1_i1 | membrane metallo-endopeptidase-like 1 | XM_011158389 | 4060 | 100 | 0 | 2.85E-05 | 0.000455 | 35.91 | 19.84 | 41 | 32.33 |
| 19 | c405735_g1_i3 | brefeldin A-inhibited guanine nucleotide-exchange protein | XM_011158872 | 5187 | 100 | 0 | 0.000132 | 0.012384 | 29.20 | 11.16 | 33 | 18 |
| 20 | c405062_g1_i1 | NADH dehydrogenase subunit 1 | KY018919 | 16300 | 100 | 0 | 0.000776 | 1.42E-09 | 22.49 | 95.65 | 23.25 | 141 |
| 21 | c404997_g1_i1 | histone H2A | XM_011168033 | 487 | 99 | 0 | 1.21E-07 | 5.92E-15 | 24.83 | 133.45 | 25.62 | 199.08 |
| 22 | c404746_g1_i2 | homeobox protein orthopedia-like | XM_011167272 | 1080 | 99 | 0 | 8.34E-05 | 0.017602 | 31.43 | 10.37 | 33.67 | 15.67 |
| 23 | c404305_g1_i1 | kinase D-interacting substrate of 220 kDa | XM_011167793 | 6375 | 100 | 0 | 0.000341 | 4.02E-05 | 16.75 | 18.80 | 18.67 | 30.33 |
| 24 | c403358_g1_i3 | glycosaminoglycan xylosylkinase | XM_011177241 | 1836 | 96 | 9E-169 | 0.000592 | 0.000213 | 23.61 | 23.00 | 28.5 | 39.75 |
| 25 | c402522_g1_i1 | anti-estrogen resistance protein 3 | XM_011169848 | 3263 | 99 | 0 | 0.001697 | 0.001331 | 13.17 | 10.89 | 14.83 | 17.5 |
| 26 | c401989_g1_i2 | membrane metallo-endopeptidase-like 1 | XM_011158389 | 4060 | 100 | 0 | 0.000123 | 0.000185 | 13.04 | 11.92 | 13.28 | 17.57 |
| 27 | c400523_g1_i3 | angiotensin-converting enzyme-like | XM_011175383 | 876 | 99 | 0 | 0.025273 | 3.21E-07 | 11.31 | 53.00 | 14 | 95.33 |
| 28 | c395740_g1_i1 | acyl-CoA Delta (11) desaturase-like | XM_011167361 | 724 | 100 | 0 | 1.71E-05 | 1.49E-05 | 17.10 | 16.73 | 17.79 | 25.21 |
| 29 | c391510_g1_i1 | venom serine protease Bi-VSP-like | XM_011175003 | 721 | 100 | 0 | 1.99E-05 | 2.91E-06 | 14.81 | 17.81 | 15.13 | 26.61 |
| 30 | c383709_g1_i1 | low-specificity L-threonine aldolase 2 | XM_011166843 | 1671 | 100 | 4E-35 | 0.036674 | 0.008799 | 10.19 | 11.95 | 11.4375 | 19.75 |
| 31 | c382591_g1_i1 | Y+L amino acid transporter 2 | XM_011166406 | 1861 | 98 | 4E-127 | 0.000592 | 0.00136 | 23.61 | 16.69 | 25 | 25.33 |
| 32 | c382567_g1_i1 | serine/arginine repetitive matrix protein 2 | XM_012681406 | 5924 | 76 | 1E-72 | 0.002454 | 0.012384 | 18.01 | 11.16 | 19.57 | 17.43 |
| 33 | c379180_g1_i1 | titin | XM_011158162 | 18888 | 99 | 0 | 8.07E-07 | 2.47E-09 | 17.88 | 32.49 | 17.73 | 46.68 |
| 34 | c374916_g1_i1 | SUN domain-containing ossification factor | XM_011701496 | 5415 | 79 | 8E-25 | 0.036674 | 0.002454 | 10.19 | 15.11 | 11.40 | 26.40 |
| 35 | c371076_g1_i1 | growth hormone-regulated TBC protein 1-A | XM_011167763 | 1877 | 99 | 4E-131 | 4.36E-06 | 0.017602 | 45.97 | 10.37 | 53.86 | 17.26 |
| 36 | c358206_g1_i1 | kinesin-like protein KIF20A | XM_011159451 | 706 | 99 | 0 | 0.001024 | 0.006311 | 21.37 | 12.74 | 25.67 | 22 |
| 37 | c343962_g2_i1 | endoplasmic reticulum aminopeptidase 1 | XM_011157586 | 6025 | 99 | 4E-87 | 0.017602 | 0.012384 | 12.42 | 11.16 | 13.29 | 17.14 |
| 38 | c197996_g1_i1 | histone H4 | XM_011157966 | 599 | 100 | 0 | 1.86E-06 | 5.28E-11 | 12.72 | 36.69 | 12.61 | 53.04 |
| 39 | c184908_g1_i1 | GTPase-activating protein CdGAPr | XR_850611 | 10316 | 100 | 5E-178 | 0.025273 | 0.017602 | 11.31 | 10.37 | 12.93 | 17.4 |
| 40 | c173171_g1_i1 | Histone H2B-like | XM_011157967 | 487 | 100 | 0 | 4.2E-05 | 6.23E-16 | 13.58 | 167.14 | 14.04 | 251.87 |
| 41 | c140434_g1_i1 | potassium voltage-gated channel subfamily H member 8 | XM_011164661 | 5399 | 100 | 0 | 0.00136 | 0.008799 | 20.25 | 11.95 | 23.6 | 19.8 |
| 42 | c127029_g1_i2 | long-chain fatty acid transport protein 1 | XM_011157045 | 2293 | 100 | 4E-59 | 0.025273 | 0.002454 | 11.31 | 15.11 | 12.83 | 25.44 |

Table S8.

| **No** | **Contig** | **Gene** | **Gene bank accession number** | **Target length** | **Identites (%)** | **e-value** | **Corrected *P*-value** | | | **Log_2_FC** | | | **FPKM** | | |
| --- | --- | --- | --- | --- | --- | --- | --- | --- | --- | --- | --- | --- | --- | --- | --- |
|  |  |  |  |  |  |  | T10/T30 | T20/T30 | T40/T30 | T10/T30 | T20/T30 | T40/T30 | T10/T30 | T20/T30 | T40/T30 |
| 1 | c416098_g1_i1 | myosin-IXa | XM_011166752 | 3540 | 100 | 2E-83 | 0.00182 | 0.001152 | 0.008799 | 19.13276 | 17.26392 | 11.9488 | 21.21 | 12.86 | 19.29 |
| 2 | c413076_g2_i2 | aromatic-L-amino-acid decarboxylase-like | XM_011163525 | 2087 | 98 | 1E-99 | 0.036674 | 4.5E-06 | 3.51E-05 | 10.18694 | 40.1029 | 29.31989 | 11.11 | 29.78 | 47.5 |
| 3 | c412792_g2_i1 | fringe glycosyltransferase | XM_011169756 | 2273 | 100 | 0 | 0.000455 | 8.9E-05 | 0.006311 | 24.7239 | 27.25597 | 12.7384 | 29 | 21 | 21 |
| 4 | c411453_g2_i1 | cGMP-dependent protein kinase | XM_011160965 | 3184 | 100 | 7E-80 | 1.02E-09 | 0.000808 | 0.000351 | 117.5368 | 18.69135 | 20.63435 | 122.17 | 12.83 | 30.67 |
| 5 | c410115_g1_i1 | Lethal (2) giant larvae protein homolog 1 | XM_011170098 | 4470 | 100 | 1E-62 | 0.004567 | 0.003595 | 0.004567 | 15.77808 | 14.40905 | 13.52799 | 18.67 | 11.33 | 23 |
| 6 | c406492_g2_i3 | afadin | XM_012677511 | 9826 | 78 | 0 | 0.002454 | 0.036674 | 1.9E-06 | 18.01453 | 11.55417 | 42.743 | 22.5 | 9.5 | 75.5 |
| 7 | c405854_g1_i1 | membrane metallo-endopeptidase-like 1 | XM_011158389 | 4060 | 100 | 0 | 2.85E-05 | 0.002428 | 0.000455 | 35.90618 | 15.83648 | 19.84475 | 41 | 12 | 32.33 |
| 8 | c405735_g1_i3 | brefeldin A-inhibited guanine nucleotide-exchange protein 1 | XM_011158872 | 5187 | 100 | 0 | 0.000132 | 0.000411 | 0.012384 | 29.19681 | 21.54623 | 11.15921 | 33 | 16 | 18 |
| 9 | c405062_g1_i1 | NADH dehydrogenase subunit 1 | KY018919 | 16300 | 100 | 0 | 0.000776 | 0.000808 | 1.42E-09 | 22.48744 | 18.69135 | 95.64586 | 23.25 | 12.75 | 141 |
| 10 | c404746_g1_i2 | homeobox protein orthopedia-like | XM_011167272 | 1080 | 99 | 0 | 8.34E-05 | 1.42E-05 | 0.017602 | 31.43326 | 34.39315 | 10.36961 | 33.67 | 24.33 | 15.67 |
| 11 | c404305_g1_i1 | kinase D-interacting substrate of 220 kDa | XM_011167793 | 6375 | 100 | 0 | 0.000341 | 0.000213 | 4.02E-05 | 16.74554 | 16.90774 | 18.80444 | 18.67 | 12.33 | 30.33 |
| 12 | c382591_g1_i1 | Y+L amino acid transporter 2 | XM_011166406 | 1861 | 98 | 4E-127 | 0.000592 | 0.00016 | 0.00136 | 23.60567 | 24.4011 | 16.68637 | 25 | 17.17 | 25.33 |
| 13 | c371076_g1_i1 | growth hormone-regulated TBC protein 1-A | XM_011167763 | 1877 | 99 | 4E-131 | 4.36E-06 | 0.001152 | 0.017602 | 45.97023 | 17.26392 | 10.36961 | 53.86 | 13.43 | 17.26 |

Table S9.

| **No** | **Contig** | **Gene** | **Target length** | **Identites (%)** | **e-value** | **Corrected *P*-value** | **Gene bank accession number** | **Fold change** | **FPKM** | |
| --- | --- | --- | --- | --- | --- | --- | --- | --- | --- | --- |
|  |  |  |  |  |  |  |  |  | T10 | T30 |
| 1 | c407973_g1_i1 | putative membrane protein | 3098 | 0 | 99 | 4.46E-45 | BAO87487.1 | 41971.1 | 9270.7 | 0.22 |
| 2 | c375063_g1_i1 | dual oxidase | 4819 | 0 | 99 | 2.6E-09 | XM_011157698 | 106.35 | 7.58 | 0.06 |
| 3 | c405143_g1_i2 | tRNA modification GTPase GTPBP3 | 5004 | 1E-85 | 100 | 3.11E-09 | XM_011166471 | 105.24 | 2.19 | 0.02 |
| 4 | c452006_g1_i1 | tumor necrosis factor receptor | 1567 | 0 | 99 | 2.8E-07 | XM_014623007 | 63.9 | 8.86 | 0.12 |
| 5 | c425690_g1_i1 | protein Malvolio | 1870 | 9E-150 | 99 | 3.21E-07 | XM_011158972 | 57.2 | 2.82 | 0.04 |
| 6 | c92359_g1_i1 | presenilin-1 | 2122 | 1E-91 | 100 | 1.2E-09 | XM_011161884 | 61.4 | 4.68 | 0.07 |
| 7 | c406820_g2_i1 | Solenopsis invicta protein quiver-like | 4068 | 2E-177 | 100 | 7.52E-07 | XM_011157571 | 57.15 | 4.25 | 0.07 |
| 8 | c411596_g2_i1 | repeat-containing protein 66-like | 2248 | 0 | 100 | 6.87E-10 | XM_011170496 | 53.23 | 2.7 | 0.05 |
| 9 | c409025_g1_i1 | protein lethal (2) denticleless | 2259 | 3E-160 | 96 | 1.38E-06 | XM_011163856 | 52.68 | 3.72 | 0.06 |
| 10 | c40334_g1_i1 | mitochondrial carrier homolog 1-like | 2107 | 1E-138 | 100 | 1.9E-06 | XM_011163645 | 51.56 | 2.52 | 0.04 |
| 11 | c247381_g1_i1 | Quadriceps-6-phosphofructo-2-kinase/fructose-2,6-bisphosphatase-like | 1661 | 8E-69 | 99 | 4.36E-06 | XM_014619992 | 45.97 | 5.13 | 0.1 |
| 12 | c456842_g1_i1 | single-minded homolog 2 | 5660 | 8E-171 | 99 | 1.07E-05 | XM_011164264 | 41.5 | 10.16 | 0.22 |
| 13 | c404098_g3_i2 | QM6a predicted protein | 2837 | 9E-150 | 100 | 5.47E-08 | XM_006961454 | 41.16 | 8.94 | 0.21 |
| 14 | c175694_g1_i1 | RNA-binding protein cabeza | 1524 | 8E-69 | 99 | 2.46E-08 | XM_011163865 | 36.58 | 3.94 | 0.1 |
| 15 | c410285_g1_i1 | CD63 antigen-like | 1467 | 3E-73 | 100 | 1.61E-09 | XM_011171872 | 36.36 | 5.27 | 0.14 |
| 16 | c409037_g1_i1 | pyruvate carboxylase, mitochondrial | 3147 | 0 | 100 | 5.59E-11 | XM_011157015 | 35.92 | 10.45 | 0.29 |
| 17 | c395697_g1_i1 | leucine-zipper-like transcriptional regulator 1 | 4499 | 0 | 100 | 3.51E-05 | XM_011160804 | 34.79 | 1.7 | 0.04 |
| 18 | c408285_g1_i1 | WD repeat-containing protein CG11141 | 471 | 0 | 95 | 3.51E-05 | XM_011157516 | 34.79 | 0.98 | 0.03 |
| 19 | c412910_g9_i1 | isopentenyl-diphosphate Delta-isomerase 1 | 5840 | 0 | 99 | 2.97E-09 | XM_011175004 | 34.07 | 5.19 | 0.15 |
| 20 | c107923_g1_i1 | transmembrane protein 245 | 1254 | 0 | 95 | 5.36E-05 | XM_012673614 | 33.67 | 1.66 | 0.04 |
| 21 | c401185_g1_i1 | Usher syndrome type-1G protein homolog | 1523 | 0 | 100 | 3.21E-07 | XM_011174581 | 33.42 | 4.90 | 0.14 |
| 22 | c401193_g1_i2 | protein gustavus | 990 | 3E-48 | 78 | 6.67E-05 | XM_018444985 | 32.55 | 4.02 | 0.11 |
| 23 | c399167_g2_i1 | protein real-time-like | 4937 | 1E-35 | 94 | 6.67E-05 | XM_011177085 | 32.55 | 8.37 | 0.23 |
| 24 | c408336_g1_i2 | synapsin | 14925 | 0 | 99 | 1.2E-09 | XM_011158270 | 31.82 | 6.82 | 0.21 |
| 25 | c406600_g3_i2 | actin, clone 403-like | 4024 | 6E-162 | 100 | 5.62E-07 | XM_011169545 | 31.64 | 5.35 | 0.16 |
| 26 | c408775_g1_i1 | histone H1A, sperm-like | 1581 | 7E-146 | 100 | 1.23E-08 | XM_011164894 | 31.53 | 4.73 | 0.15 |
| 27 | c411596_g3_i2 | WD repeat-containing protein 66-like | 1581 | 0 | 100 | 4.33E-10 | XM_011170496 | 31.52 | 13.36 | 0.43 |
| 28 | c407395_g2_i1 | cytochrome P450 6k1-like | 2724 | 1E-122 | 100 | 4.07E-08 | 105206904 | 30.5 | 3.09 | 0.1 |
| 29 | c409183_g2_i3 | inactive rhomboid protein 1 | 547 | 5E-30 | 100 | 5.68E-10 | XM_011158868 | 29.48 | 7.28 | 0.25 |
| 30 | c106289_g1_i1 | transmembrane protein C9orf91 homolog | 4937 | 0 | 100 | 0.000132 | XM_011158245 | 29.20 | 2.6 | 0.08 |
| 31 | c403826_g1_i1 | latrophilin Cirl-like | 1719 | 0 | 87 | 0.000132 | XM_011157580 | 29.20 | 4.93 | 0.15 |
| 32 | c398764_g1_i1 | putative serine/threonine-protein kinase PRKY | 358 | 6E-64 | 87 | 2.04E-07 | XM_011173077 | 28.87 | 9.2 | 0.31 |
| 33 | c404341_g2_i3 | cysteine-rich motor neuron 1 protein-like | 4548 | 0 | 100 | 7.42E-08 | XM_011160425 | 28.67 | 3.32 | 0.11 |
| 34 | c386807_g1_i2 | protein wings apart-like | 9309 | 8E-118 | 100 | 1.38E-06 | XM_018542833 | 28.06 | 1.94 | 0.07 |
| 35 | c403862_g1_i1 | COP9 signalosome complex subunit 6 | 1778 | 9E-150 | 100 | 8.86E-08 | XM_011172999 | 28.05 | 5.85 | 0.20 |
| 36 | c406202_g1_i1 | serine/threonine-protein kinase Nek5-like | 5891 | 2E-17 | 100 | 9.02E-06 | XM_011169733 | 27.47 | 6.77 | 0.23 |
| 37 | c413191_g6_i2 | major antigen-like | 1045 | 0 | 99 | 5.13E-07 | XM_011173517 | 26.02 | 8.62 | 0.32 |
| 38 | c284906_g1_i1 | voltage-dependent T-type calcium channel subunit alpha-1G | 11912 | 8E-72 | 85 | 0.000273 | XM_011158609 | 25.84 | 3.71 | 0.13 |
| 39 | c447409_g1_i1 | transcription factor mef2A-like | 6072 | 0 | 99 | 0.000273 | XM_011163996 | 25.84 | 2.61 | 0.09 |
| 40 | c404245_g2_i1 | peptidyl-prolyl cis-trans isomerase-like | 3788 | 1E-48 | 83 | 8.4E-07 | XM_011165809 | 24.81 | 1.86 | 0.07 |
| 41 | c14655_g1_i1 | tubulin glycylase 3A | 9837 | 0 | 100 | 2.02E-05 | XM_011161947 | 24.49 | 4.42 | 0.17 |
| 42 | c423254_g1_i1 | ran GTPase-activating protein 1 | 2107 | 8E-49 | 100 | 2.39E-05 | XM_011159666 | 23.89 | 2.97 | 0.12 |
| 43 | c405878_g2_i1 | laminin subunit alpha-1 | 2419 | 4E-111 | 100 | 2.06E-08 | XM_018517448 | 23.78 | 4.86 | 0.21 |
| 44 | c447481_g1_i1 | cuticle protein 19.8-like | 3506 | 1E-13 | 93 | 0.000592 | XM_012666484 | 23.61 | 3.04 | 0.11 |
| 45 | c377683_g1_i1 | E3 ubiquitin-protein ligase MYCBP2-like | 4249 | 1E-104 | 100 | 0.000592 | XM_011177800 | 23.60 | 2.20 | 0.08 |
| 46 | c396785_g2_i1 | nucleoporin p54 | 5952 | 0 | 100 | 0.000592 | XM_011170486 | 23.60 | 0.7 | 0.03 |
| 47 | c396466_g1_i1 | kinesin-like protein unc-104 | 2399738 | 0 | 100 | 1.83E-07 | XM_018548445 | 23.59 | 5.88 | 0.25 |
| 48 | c411522_g2_i1 | Soap Lake #7 | 3926 | 0 | 99 | 5.31E-08 | CP019915 | 23.04 | 3.07 | 0.13 |
| 49 | c400642_g1_i1 | RNA helicase armi | 4259 | 0 | 99 | 2.58E-07 | XM_011177698 | 22.85 | 3.17 | 0.14 |
| 50 | c394590_g1_i2 | sodium/potassium-transporting ATPase subunit beta-2-like | 699 | 0 | 99 | 1.61E-06 | XR_001931667 | 22.78 | 5.83 | 0.25 |
| 51 | c413360_g4_i1 | embryonic polarity protein dorsal | 2716 | 0 | 100 | 6.22E-07 | XM_012019151 | 22.51 | 4.42 | 0.19 |
| 52 | c129929_g1_i1 | protein phosphatase 1B | 1447 | 4E-86 | 99 | 0.000776 | XM_011064095 | 22.49 | 1.88 | 0.07 |
| 53 | c412868_g4_i1 | putative golgin subfamily A member 6-like protein 6 | 988 | 7E-123 | 100 | 1.84E-06 | 105667571 | 22.37 | 10.43 | 0.45 |
| 54 | c372556_g1_i1 | diacylglycerol kinase 1 | 1050 | 0.000005 | 83 | 4.8E-05 | XM_011167221 | 22.10 | 3.69 | 0.16 |
| 55 | c385846_g1_i1 | ARSEF 2860 DDHD domain-containing protein | 3281 | 0 | 99 | 2.41E-06 | XM_008598457 | 21.97 | 4.28 | 0.19 |
| 56 | c392942_g1_i1 | ribosome biogenesis protein BRX1 homolog | 456 | 3E-132 | 91 | 5.76E-05 | XM_011162833 | 21.50 | 2.02 | 0.09 |
| 57 | c442774_g1_i1 | nucleolar complex protein 2 homolog | 1733 | 7E-165 | 100 | 5.76E-05 | XM_011177819 | 21.50 | 3.69 | 0.16 |
| 58 | c169221_g1_i1 | RNA-directed DNA polymerase from mobile element jockey-like | 2550 | 0 | 97 | 0.001024 | XM_011170549 | 21.37 | 3.22 | 0.13 |
| 59 | c394375_g2_i1 | sarcoplasmic calcium-binding protein 1 | 8813524 | 0 | 89 | 0.001024 | XM_011169115 | 21.37 | 3.56 | 0.15 |
| 60 | c341844_g1_i1 | EHB-P0848 #02 16S ribosomal RNA gene | 9042 | 1E-75 | 87 | 1.63E-08 | KU978308 | 21.20 | 43.34 | 2.05 |
| 61 | c402739_g1_i1 | ras-related protein Rab-40B | 3290 | 2E-180 | 85 | 3.19E-06 | XM_011158592 | 21.16 | 1.89 | 0.09 |
| 62 | c441596_g1_i1 | homeobox protein cut | 11043 | 0 | 98 | 0.00136 | XM_011164784 | 20.25 | 2.0 | 0.09 |
| 63 | c406205_g2_i3 | CUGBP Elav-like family member 4 | 6790 | 3E-42 | 100 | 3.41E-08 | XM_011169467 | 19.90 | 7.21 | 0.36 |
| 64 | c383576_g2_i1 | lysosomal Pro-X carboxypeptidase | 1661 | 6E-69 | 84 | 5.71E-06 | XM_011164621 | 19.53 | 2.82 | 0.14 |
| 65 | c86326_g1_i1 | E3 ubiquitin-protein ligase hulA | 3014 | 5E-140 | 100 | 0.00182 | XM_022620997 | 19.13 | 1.86 | 0.09 |
| 66 | c405044_g1_i1 | eukaryotic translation initiation factor 3 subunit B | 2659 | 0 | 99 | 0.00182 | XM_011177915 | 19.13 | 1.24 | 0.06 |
| 67 | c411274_g1_i3 | protein SERAC1 | 3854 | 0 | 100 | 7.62E-07 | XM_011169402 | 18.91 | 4.42 | 0.23 |
| 68 | c410351_g3_i1 | tetraspanin-1 | 1565 | 3E-67 | 83 | 5.47E-07 | XM_011175080 | 18.59 | 9.08 | 0.49 |
| 69 | c381942_g1_i2 | protogenin-like | 2399738 | 0 | 99 | 0.00015 | XM_011160795 | 18.53 | 1.68 | 0.09 |
| 70 | c398612_g1_i1 | UPF0704 protein C6orf165 homolog | 1336 | 1E-161 | 83 | 1.72E-06 | XM_011176112 | 18.38 | 2.66 | 0.14 |
| 71 | c400319_g1_i1 | hexaprenyldihydroxybenzoate methyltransferase, mitochondrial-like | 2217 | 1E-64 | 100 | 1.72E-06 | XM_011160316 | 18.38 | 2.05 | 0.11 |
| 72 | c409420_g1_i1 | gamma-glutamylcyclotransferase CG2811 | 2452 | 0 | 100 | 3.02E-08 | XM_011161965 | 18.05 | 11.92 | 0.67 |
| 73 | c412780_g1_i1 | membrane-associated guanylate kinase | 4803686 | 6E-164 | 81 | 0.002454 | XM_018448842 | 18.01 | 1.78 | 0.09 |
| 74 | c178564_g1_i1 | solute carrier family 15 member 1 | 2481 | 1E-111 | 82 | 0.002454 | XM_011174105 | 18.01 | 2.13 | 0.11 |
| 75 | c410780_g2_i1 | mitochondrial dicarboxylate carrier | 3075 | 0 | 99 | 0.000224 | XM_011162575 | 17.94 | 3.15 | 0.17 |
| 76 | c410373_g1_i1 | Cutibacterium acnes strain A1-14 chromosome | 3172 | 8E-105 | 78 | 7.92E-08 | CP013693 | 17.41 | 14.91 | 0.86 |
| 77 | c250653_g1_i1 | longitudinals lacking protein, isoform G | 1061 | 0 | 100 | 7.69E-09 | XM_011162192 | 17.37 | 64.75 | 3.78 |
| 78 | c382916_g1_i1 | Uncultured bacterium 213AG01013 gene for 16S rRNA | 3430 | 6E-152 | 100 | 1.08E-07 | LC355665 | 17.19 | 26.63 | 1.56 |
| 79 | c173518_g1_i2 | pupal cuticle protein Edg-91-like | 1617 | 0 | 99 | 1.88E-08 | XM_011162035 | 17.13 | 15.69 | 0.93 |
| 80 | c410907_g2_i1 | ribosomal protein S13, S15 family | 3985 | 0 | 99 | 6.6E-06 | XM_018322315 | 16.97 | 5.97 | 0.35 |
| 81 | c58820_g1_i1 | neurogenic protein mastermind | 3254 | 0 | 100 | 0.003334 | XM_018189020 | 16.90 | 1.71 | 0.09 |
| 82 | c407894_g2_i1 | testis-expressed sequence 9 protein | 1323 | 0 | 99 | 0.000341 | XM_011159442 | 16.75 | 3.47 | 0.20 |
| 83 | c411115_g2_i2 | voltage-dependent T-type calcium channel subunit alpha-1G | 1914 | 3E-119 | 100 | 5.62E-08 | XM_011158609 | 16.65 | 4.86 | 0.30 |
| 84 | c370067_g1_i1 | protein lifeguard 1 | 1109 | 1E-117 | 100 | 1.51E-07 | XM_011163381 | 16.60 | 6.70 | 0.41 |
| 85 | c411909_g4_i2 | facilitated trehalose transporter Tret1-2 homolog | 848 | 0 | 100 | 1.27E-07 | XM_011163248 | 16.59 | 4.82 | 0.29 |
| 86 | c407264_g1_i1 | CBS 148.51 40S ribosomal protein S18 | 2399738 | 0 | 99 | 4.53E-06 | XM_001229651 | 16.40 | 6.22 | 0.37 |
| 87 | c408362_g1_i1 | U6 snRNA-associated Sm-like protein LSm4 | 1350 | 0 | 100 | 2.85E-07 | XM_011166787 | 16.38 | 4.64 | 0.28 |
| 88 | c403316_g2_i1 | putative leucine-rich repeat-containing protein DDB_G0290503 | 2157 | 6E-65 | 99 | 0.000424 | XM_011167568 | 16.15 | 1.68 | 0.10 |
| 89 | c422188_g1_i1 | esterase SG1-like | 3996 | 6E-139 | 100 | 0.000424 | XM_011177464 | 16.15 | 2.71 | 0.16 |
| 90 | c438858_g1_i1 | protein tipE | 1707 | 0 | 99 | 0.000424 | XM_011160305 | 16.15 | 1.65 | 0.10 |
| 91 | c397784_g1_i1 | leucine carboxyl methyltransferase 1 | 878 | 0 | 100 | 2.83E-05 | XM_011168180 | 15.88 | 1.45 | 0.09 |
| 92 | c414140_g1_i1 | glycine receptor subunit alpha-2-like | 2422 | 0 | 100 | 0.004567 | XM_012018897 | 15.78 | 1.57 | 0.09 |
| 93 | c451160_g1_i1 | zinc finger protein 608-like | 2702 | 0 | 100 | 0.004567 | XM_012016915 | 15.78 | 1.70 | 0.10 |
| 94 | c174144_g1_i1 | beta-1,3-galactosyltransferase 5-like | 2763 | 8E-60 | 85 | 0.004567 | XM_011157841 | 15.78 | 1.37 | 0.08 |
| 95 | c386972_g1_i1 | odorant receptor 22c-like | 2928 | 0 | 99 | 0.004567 | XM_011163972 | 15.78 | 1.28 | 0.07 |
| 96 | c400996_g1_i1 | lysosomal-trafficking regulator | 4433 | 0 | 99 | 0.004567 | XM_011167308 | 15.78 | 0.97 | 0.06 |
| 97 | c403411_g1_i2 | platelet-derived growth factor subunit A-like | 1872 | 0 | 99 | 0.004567 | XM_011170646 | 15.78 | 0.44 | 0.03 |
| 98 | c410926_g3_i1 | homeotic protein female sterile-like | 4645 | 2E-33 | 100 | 0.004567 | XM_011157243 | 15.78 | 1.55 | 0.09 |
| 99 | c455230_g1_i1 | synaptotagmin-10-like | 2136 | 0 | 99 | 0.004567 | XM_011162408 | 15.78 | 2.07 | 0.12 |
| 100 | c389696_g1_i1 | protein expanded | 1516 | 6E-64 | 97 | 6.39E-06 | XM_011165982 | 15.65 | 2.01 | 0.13 |
| 101 | c298901_g1_i1 | maternal protein tudor-like | 2730 | 8E-114 | 100 | 0.000528 | XM_011177695 | 15.55 | 3.23 | 0.20 |
| 102 | c412057_g4_i1 | choline transporter-like protein 1 | 1672 | 4E-110 | 100 | 2.83E-06 | XM_011163235 | 15.37 | 3.36 | 0.22 |
| 103 | c399434_g1_i1 | peroxisomal membrane protein 2 | 3577 | 3E-93 | 90 | 0.000661 | XM_011177123 | 14.96 | 1.71 | 0.11 |
| 104 | c412420_g2_i1 | 5-oxoprolinase | 2770 | 4E-103 | 99 | 0.000661 | XM_011157065 | 14.96 | 5.95 | 0.38 |
| 105 | c45160_g1_i1 | parkin co-regulated gene protein homolog | 4713 | 3E-180 | 100 | 0.000661 | XM_011175331 | 14.96 | 1.38 | 0.09 |
| 106 | c2447_g1_i1 | Neff EF1 guanine nucleotide exchange domain containing protein | 1035 | 0 | 96 | 0.008799 | XM_004338017 | 14.66 | 1.89 | 0.12 |
| 107 | c415378_g1_i1 | sphingomyelin phosphodiesterase 1-like | 3323 | 9E-73 | 98 | 0.008799 | XM_011175377 | 14.66 | 0.89 | 0.05 |
| 108 | c419016_g1_i1 | major antigen-like | 1695 | 5E-25 | 100 | 0.008799 | XM_011161426 | 14.66 | 1.66 | 0.10 |
| 109 | c424235_g1_i1 | GRAM domain-containing protein 1B-like | 3933432 | 0 | 99 | 0.008799 | XM_011161667 | 14.66 | 1.42 | 0.09 |
| 110 | c433933_g1_i1 | tyrosine-protein phosphatase 99A | 6425 | 1E-95 | 99 | 0.008799 | XM_011160511 | 14.66 | 1.55 | 0.09 |
| 111 | c394557_g1_i1 | midnolin-A-like | 4698 | 6E-147 | 100 | 0.000831 | XM_011177580 | 14.37 | 4.50 | 0.30 |
| 112 | c423247_g1_i1 | FTDNA 622721 haplogroup X mitochondrion | 3963 | 0 | 100 | 0.000831 | MG592700 | 14.36 | 3.78 | 0.25 |
| 113 | c403754_g1_i1 | apolipoprotein D-like | 4890 | 1E-41 | 77 | 1.22E-06 | XM_011162283 | 13.97 | 7.30 | 0.52 |
| 114 | c252257_g1_i1 | enolase-phosphatase E1-like | 4527 | 2E-172 | 86 | 8.36E-05 | XM_011171170 | 13.85 | 1.30 | 0.09 |
| 115 | c435097_g1_i1 | non-histone chromosomal protein 6 homolog | 2593 | 1E-66 | 100 | 5.16E-06 | XM_012670098 | 13.80 | 8.36 | 0.60 |
| 116 | c406059_g2_i1 | putative 60s ribosomal protein l2 protein | 2182 | 2E-153 | 100 | 0.001049 | XM_006961160 | 13.77 | 5.80 | 0.40 |
| 117 | c393914_g1_i1 | mitochondrial-processing peptidase subunit beta | 1194 | 0 | 100 | 1.16E-05 | XM_011173810 | 13.73 | 3.30 | 0.24 |
| 118 | c358837_g2_i1 | cAMP-dependent protein kinase catalytic subunit | 1443 | 2E-145 | 100 | 0.012384 | XM_011157529 | 13.54 | 1.14 | 0.08 |
| 119 | c459765_g1_i1 | doublesex and mab-3 related transcription factor 3, truncated-like | 2902 | 3E-10 | 95 | 0.012384 | XM_012682882 | 13.54 | 5.07 | 0.33 |
| 120 | c462895_g1_i1 | KN motif and ankyrin repeat domain-containing protein 2 | 1986 | 0 | 91 | 0.012384 | XM_011173822 | 13.54 | 1.61 | 0.11 |
| 121 | c79374_g1_i2 | proline-rich nuclear receptor coactivator 2-like | 1045 | 2E-98 | 100 | 0.012384 | XM_018452531 | 13.54 | 0.75 | 0.05 |
| 122 | c399155_g2_i1 | unconventional myosin-XVIIIa-like | 7316 | 5E-14 | 98 | 0.012384 | XM_011164511 | 13.54 | 3.43 | 0.23 |
| 123 | c455794_g1_i1 | transcription factor GATA-4-like | 1488 | 7E-78 | 100 | 0.012384 | XM_011172678 | 13.54 | 2.24 | 0.15 |
| 124 | c369055_g1_i1 | adhesive plaque matrix protein 2-like | 1059 | 2E-102 | 100 | 3.79E-07 | XM_011157856 | 13.51 | 10.14 | 0.76 |
| 125 | c401581_g2_i1 | cytoplasmic dynein 2 light intermediate chain 1 | 6451 | 0 | 99 | 0.001697 | XM_011157787 | 13.17 | 2.74 | 0.20 |
| 126 | c407618_g1_i1 | major urinary protein 19 (Mup19) | 2921 | 0 | 100 | 0.000123 | NM_001135127 | 13.04 | 2.68 | 0.20 |
| 127 | c371411_g1_i1 | 26S proteasome non-ATPase regulatory subunit 2-like | 1478 | 0 | 100 | 1.29E-06 | XM_011163303 | 12.88 | 4.28 | 0.34 |
| 128 | c408912_g1_i1 | transmembrane protein 35 | 2508 | 2E-36 | 88 | 6.74E-05 | XM_011159916 | 12.66 | 3.20 | 0.25 |
| 129 | c409005_g1_i2 | transcription initiation factor IIA subunit 1 | 11043 | 2E-178 | 100 | 6.99E-06 | XM_011163766 | 12.59 | 2.56 | 0.20 |
| 130 | c422593_g1_i1 | calcium-activated potassium channel slowpoke | 6525 | 0 | 99 | 0.002174 | XM_012673538 | 12.58 | 1.86 | 0.14 |
| 131 | c175961_g1_i1 | muscle M-line assembly protein unc-89 | 1897 | 3E-49 | 83 | 2.59E-05 | XM_011158167 | 12.48 | 2.22 | 0.18 |
| 132 | c135088_g1_i1 | protein NDRG3 | 1566 | 1E-115 | 86 | 0.017602 | XM_012681016 | 12.42 | 0.81 | 0.06 |
| 133 | c162384_g1_i1 | zinc finger protein rotund-like | 8704 | 0 | 100 | 0.017602 | XM_011163000 | 12.42 | 1.88 | 0.14 |
| 134 | c431944_g1_i1 | adenylate cyclase type 6 | 6467 | 1E-135 | 88 | 0.017602 | XM_011173654 | 12.42 | 2.26 | 0.16 |
| 135 | c454175_g1_i1 | aldehyde dehydrogenase, dimeric NADP-preferring-like | 2308 | 4E-60 | 84 | 0.017602 | XM_011173880 | 12.42 | 2.95 | 0.21 |
| 136 | c409023_g1_i4 | puromycin-sensitive aminopeptidase | 1505 | 0 | 100 | 4.18E-07 | XM_011166782 | 12.40 | 5.12 | 0.42 |
| 137 | c343018_g2_i1 | probable E3 ubiquitin-protein ligase RNF144A | 2755 | 5E-65 | 86 | 9.35E-05 | XM_011639489 | 12.04 | 5.28 | 0.43 |
| 138 | c377728_g1_i1 | schwannomin-interacting protein 1 | 1657 | 0 | 100 | 2.38E-05 | XM_011161528 | 11.99 | 1.28 | 0.11 |
| 139 | c78428_g1_i1 | epidermal growth factor receptor kinase substrate 8-like | 1231 | 2E-104 | 99 | 0.002801 | XM_011173841 | 11.97 | 1.42 | 0.11 |
| 140 | c389763_g1_i1 | putative glutamate synthase [NADPH] | 2962 | 6E-24 | 83 | 0.002801 | XM_011062501 | 11.98 | 2.72 | 0.22 |
| 141 | c456880_g1_i1 | cytochrome c oxidase subunit 7A1, mitochondrial-like | 1468 | 3E-179 | 100 | 0.002801 | XM_011165483 | 11.98 | 2.18 | 0.17 |
| 142 | c172501_g1_i1 | thioredoxin domain-containing protein 5 | 3845 | 0 | 100 | 2.77E-06 | XM_011161238 | 11.93 | 4.13 | 0.35 |
| 143 | c412839_g2_i1 | glucose-regulated protein (HSP70) | 920 | 0 | 100 | 5.58E-05 | XM_001227132 | 11.93 | 2.01 | 0.17 |
| 144 | c402317_g1_i1 | cyclin-dependent kinase 20 | 3093 | 0 | 99 | 0.000276 | XM_011167624 | 11.81 | 1.75 | 0.14 |
| 145 | c428278_g1_i1 | CD2 antigen cytoplasmic tail-binding protein 2 homolog | 2901 | 4E-145 | 100 | 0.000276 | XM_011172630 | 11.82 | 2.09 | 0.17 |
| 146 | c412841_g2_i1 | chromobox protein homolog 5-like | 3332 | 0 | 100 | 6.43E-05 | XM_011158904 | 11.68 | 1.99 | 0.17 |
| 147 | c356244_g1_i1 | ADP-dependent glucokinase | 1937 | 0 | 100 | 4.19E-05 | XM_011174781 | 11.65 | 2.72 | 0.23 |
| 148 | c369768_g1_i1 | F-box/WD repeat-containing protein 4-like | 5121 | 0 | 99 | 4.19E-05 | XM_011166330 | 11.65 | 1.86 | 0.16 |
| 149 | c402338_g1_i2 | JK18.2 gene for 16S ribosomal RNA | 13963 | 0 | 84 | 6.99E-06 | LC341280 | 11.46 | 15.76 | 1.38 |
| 150 | c402295_g1_i1 | 14-3-3 zeta gene | 3298 | 0 | 100 | 4.74E-05 | JN571279 | 11.44 | 3.96 | 0.34 |
| 151 | c412924_g2_i2 | Podospora anserina genomic DNA, chromosome 1 | 4563 | 0 | 100 | 7.23E-07 | FO904936 | 11.41 | 11.90 | 1.06 |
| 152 | c389578_g2_i1 | cytoskeleton-associated protein 5 | 4287 | 0 | 100 | 0.000341 | XM_011158797 | 11.41 | 3.30 | 0.28 |
| 153 | c406967_g1_i1 | probable serine/threonine-protein kinase kinX | 1537 | 0 | 99 | 0.003627 | XM_011162704 | 11.38 | 1.37 | 0.11 |
| 154 | c405083_g1_i1 | solute carrier family 26 member 6 | 799 | 1E-83 | 89 | 0.003627 | XM_011162014 | 11.38 | 4.46 | 0.37 |
| 155 | c107512_g1_i1 | facilitated trehalose transporter Tret1 |  |  |  | 0.025273 | XM_011159872 | 11.30 | 3.50 | 0.28 |
| 156 | c92430_g1_i1 | tetraspanin-1 | 1622 | 5E-59 | 91 | 0.025273 | XM_011175080 | 11.30 | 3.27 | 0.26 |
| 157 | c369861_g1_i2 | E3 ubiquitin-protein ligase RNF19A-like | 952 | 0 | 100 | 0.025273 | XM_011647308 | 11.30 | 1.09 | 0.09 |
| 158 | c395999_g2_i2 | dynein light chain 4, axonemal | 7096 | 1E-100 | 100 | 0.025273 | XM_012683746 | 11.30 | 0.77 | 0.06 |
| 159 | c434000_g1_i1 | meiosis-specific nuclear structural protein 1-like | 1760 | 6E-27 | 100 | 0.025273 | 105202950 | 11.30 | 4.12 | 0.33 |
| 160 | c390638_g1_i1 | tumor necrosis factor alpha-induced protein 8-like protein | 5183 | 4E-100 | 89 | 0.025273 | XM_012686080 | 11.30 | 1.16 | 0.09 |
| 161 | c393875_g1_i3 | protein FAM154A-like | 1833 | 0 | 100 | 6.1E-05 | XM_011157056 | 11.23 | 2.33 | 0.21 |
| 162 | c407930_g2_i1 | transposable element Tc3 transposase | 1932 | 3E-120 | 100 | 0.000185 | XM_020029790 | 11.12 | 1.81 | 0.16 |
| 163 | c386051_g1_i1 | RNA-directed DNA polymerase from mobile element jockey-like | 2214 | 0 | 100 | 0.000185 | 106721443 | 11.12 | 2.16 | 0.19 |
| 164 | c412791_g7_i1 | methyltransferase-like protein 25 | 822 | 4E-89 | 99 | 0.000185 | XM_011169567 | 11.12 | 3.19 | 0.28 |
| 165 | c344153_g1_i1 | probable peptide chain release factor C12orf65 homolog, mitochondrial | 2108 | 0 | 100 | 4.61E-05 | XM_011175914 | 11.09 | 2.00 | 0.18 |
| 166 | c63791_g1_i1 | ultra-conserved element locus uce-1473 genomic sequence | 873 | 6E-166 | 100 | 0.000424 | KM432600 | 11.01 | 3.05 | 0.27 |
| 167 | c393714_g1_i1 | probable DNA mismatch repair protein Msh6 | 1244 | 0 | 100 | 0.000424 | XM_011175400 | 11.01 | 1.21 | 0.11 |
| 168 | c412767_g3_i1 | activating signal cointegrator 1 complex subunit 3 | 2914 | 3E-12 | 97 | 0.000424 | XM_011174611 | 11.01 | 2.93 | 0.26 |
| 169 | c403572_g1_i1 | aspartic protease | 4937 | 0 | 100 | 0.000115 | XM_003654279 | 10.94 | 2.20 | 0.20 |
| 170 | c377861_g1_i1 | enolase-phosphatase E1-like | 6366 | 0 | 100 | 0.000115 | XM_011171170 | 10.94 | 2.26 | 0.20 |
| 171 | c411510_g1_i1 | strain A1-14 chromosome | 6670 | 6E-21 | 78 | 1E-06 | CP013693 | 10.87 | 12.92 | 1.21 |
| 172 | c374064_g1_i1 | motile sperm domain-containing protein 2-like | 2053 | 2E-140 | 91 | 0.004724 | XM_011170165 | 10.79 | 8.62 | 0.76 |
| 173 | c47145_g1_i2 | transcription initiation factor TFIID subunit 12 | 2585 | 4E-10 | 87 | 0.004724 | XM_011169226 | 10.79 | 2.19 | 0.19 |
| 174 | c417775_g1_i1 | E2/E3 hybrid ubiquitin-protein ligase UBE2O | 2367 | 0 | 100 | 0.004724 | XM_011164638 | 10.79 | 3.27 | 0.29 |
| 175 | c435250_g1_i1 | patj homolog | 1905 | 0 | 100 | 0.000528 | XM_011162056 | 10.60 | 5.89 | 0.54 |
| 176 | c396882_g1_i1 | atypical protein kinase C | 4292 | 0 | 100 | 4.17E-06 | XM_018488559 | 10.58 | 8.41 | 0.80 |
| 177 | c375524_g1_i1 | peroxisomal hydratase-dehydrogenase-epimerase-like | 3182 | 0 | 98 | 0.000156 | XM_011163752 | 10.44 | 6.05 | 0.57 |
| 178 | c273746_g1_i1 | pyruvate carboxylase, mitochondrial | 3147 | 0 | 100 | 0.000103 | XM_011157014 | 10.40 | 2.57 | 0.25 |
| 179 | c407752_g2_i4 | regulator of G-protein signaling 7-binding protein-like | 5533 | 2E-148 | 100 | 0.000103 | XM_011157520 | 10.40 | 1.74 | 0.17 |
| 180 | c412461_g13_i1 | myosin-11-like | 531 | 0 | 100 | 2.96E-06 | XM_018542728 | 10.32 | 4.12 | 0.40 |
| 181 | c408737_g2_i1 | DDB1- and CUL4-associated factor 8-like | 2306 | 4E-89 | 100 | 3.13E-06 | XM_011163968 | 10.29 | 6.09 | 0.60 |
| 182 | c398616_g1_i1 | UDP-N-acetylglucosamine--peptide N-acetylglucosaminyltransferase | 3882 | 0 | 99 | 4.77E-06 | XM_012682115 | 10.20 | 10.90 | 1.08 |
| 183 | c408798_g1_i1 | strain A1-14 chromosome | 4296 | 0 | 100 | 5.27E-06 | CP013693 | 10.20 | 12.68 | 1.26 |
| 184 | c448748_g1_i1 | eukaryotic translation initiation factor 1 alpha 1 | 2337 | 3E-84 | 100 | 0.000182 | XP_001745655 | 10.20 | 3.89 | 0.38 |
| 185 | c403340_g1_i1 | neurobeachin | 1461 | 2E-44 | 85 | 0.000182 | XM_011162434 | 10.20 | 2.07 | 0.20 |
| 186 | c401586_g1_i2 | zinc finger BED domain-containing protein 1 | 4331 | 2E-166 | 83 | 0.000661 | XM_011338418 | 10.19 | 1.41 | 0.14 |
| 187 | c406273_g1_i1 | esterase E4-like | 12008 | 1E-111 | 98 | 0.000661 | XM_011707183 | 10.19 | 10.43 | 0.99 |
| 188 | c133338_g1_i1 | rap1 GTPase-activating protein 1-like | 2114 | 7E-51 | 100 | 0.006189 | XM_018504440 | 10.19 | 2.90 | 0.27 |
| 189 | c388979_g2_i1 | serine/arginine-rich splicing factor 2 | 453 | 2E-55 | 76 | 0.006189 | XR_850931 | 10.20 | 1.81 | 0.17 |
| 190 | c404138_g2_i1 | nephrin |  |  |  | 0.006189 | XM_012371016 | 10.19 | 2.68 | 0.25 |
| 191 | c438778_g1_i1 | transcription factor Sox-3 | 1627 | 1E-11 | 86 | 0.006189 | XM_011163231 | 10.19 | 7.63 | 0.71 |
| 192 | c403999_g2_i1 | myoneurin-like | 1854 | 0 | 99 | 0.036674 | XM_012025743 | 10.19 | 1.65 | 0.15 |
| 193 | c412470_g1_i1 | lysosomal alpha-glucosidase-like | 3834 | 8E-48 | 76 | 0.036674 | XM_011162212 | 10.19 | 0.92 | 0.08 |
| 194 | c459130_g1_i1 | protein Skeletor, isoforms B/C | 4420 | 4E-10 | 91 | 0.036674 | XM_012673072 | 10.19 | 2.97 | 0.26 |
| 195 | c413477_g1_i1 | putative nuclease HARBI1 | 1885 | 2E-180 | 100 | 0.036674 | 109862298 | 10.19 | 2.51 | 0.22 |
| 196 | c8897_g1_i1 | zinc finger protein ZFPM1 | 2411 | 2E-104 | 80 | 0.036674 | XM_011169882 | 10.19 | 2.39 | 0.21 |
| 197 | c151835_g1_i1 | ataxin-7-like protein 2 | 4735 | 2E-83 | 100 | 0.036674 | XM_011173825 | 10.19 | 1.61 | 0.14 |
| 198 | c160004_g1_i1 | cell wall protein DAN4 | 4074 | 0 | 100 | 0.036674 | XM_011167970 | 10.19 | 1.52 | 0.13 |
| 199 | c423169_g1_i1 | brain tumor protein | 809 | 9E-73 | 100 | 0.036674 | XM_011157530 | 10.19 | 1.29 | 0.11 |
| 200 | c458719_g1_i1 | protein furry-like | 3411 | 0 | 91 | 0.036674 | XM_011174964 | 10.19 | 0.99 | 0.09 |
| 201 | c430523_g1_i1 | SGT1 homolog ecdysoneless | 5793 | 0 | 99 | 1.73E-06 | XM_011177706 | 10.12 | 35.44 | 3.55 |
| 202 | c393791_g1_i1 | CUE domain-containing protein 2 | 16572 | 0 | 100 | 1.53E-05 | XM_011168289 | 10.10 | 1.83 | 0.18 |
| 203 | c5572_g1_i1 | porphobilinogen deaminase-like | 1986 | 1E-91 | 100 | 4.21E-05 | XM_011172927 | 10.07 | 4.59 | 0.46 |

Table S10.

| **No** | **Contig** | **Gene** | **Target length** | **Identites (%)** | **e-value** | **Corrected *P-*value** | **Gene bank accession number** | **Fold change** | FPKM | |
| --- | --- | --- | --- | --- | --- | --- | --- | --- | --- | --- |
|  |  |  |  |  |  |  |  |  | T20 | T30 |
| 1 | c410975_g1_i1 | venom allergen 4 | 399 | 98 | 0 | 2.04E-07 | 105205100 | 32.91 | 4.59 | 0.2 |
| 2 | c355863_g1_i1 | zinc finger BED domain-containing protein 1-like | 3660 | 90 | 9E-09 | 4.05E-07 | XM_012028726 | 30.89 | 4.08 | 0.19 |
| 3 | c377608_g1_i1 | hemoglobin, gamma A, mRNA | 612 | 100 | 0 | 3.9E-05 | BC020719 | 30.11 | 1.51 | 0.07 |
| 4 | c410199_g1_i4 | hymenoptaecin | 2657 | 97 | 0 | 1.56E-10 | 105196795 | 28.71 | 54.39 | 2.91 |
| 5 | c412852_g4_i1 | DNA-(apurinic or apyrimidinic site) lyase | 865 | 100 | 0 | 5.1E-05 | 105193701 | 28.68 | 3.80 | 0.17 |
| 6 | c411702_g1_i3 | muscle M-line assembly protein unc-89 | 9837 | 100 | 0 | 8.1E-08 | XM_011158167 | 25.95 | 3.11 | 0.18 |
| 7 | c402950_g1_i1 | phosphatidylserine synthase 1 | 1848 | 100 | 0 | 4.92E-06 | XM_011158390 | 23.05 | 1.56 | 0.10 |
| 8 | c352703_g1_i1 | trifunctional purine biosynthetic protein adenosine-3 | 3341 | 100 | 2E-47 | 8.34E-05 | XM_011160820 | 19.20 | 0.94 | 0.07 |
| 9 | c456297_g1_i1 | zinc finger and BTB domain-containing protein 49-like | 7508 | 84 | 1E-70 | 0.000808 | XM_011697405 | 18.69 | 2.08 | 0.15 |
| 10 | c399384_g1_i1 | protein RER1 | 902 | 100 | 0 | 0.000808 | XM_011157853 | 18.69 | 0.56 | 0.04 |
| 11 | c457143_g1_i1 | abaecin | 445 | 100 | 0 | 3.38E-09 | XR_850725 | 18.52 | 75.22 | 6.30 |
| 12 | c411466_g3_i1 | transportin-1 | 2573 | 100 | 0 | 0.000132 | XM_011167983 | 17.67 | 1.95 | 0.16 |
| 13 | c183421_g1_i1 | netrin-B | 7461 | 82 | 1E-26 | 0.001152 | XM_012318362 | 17.26 | 1.13 | 0.09 |
| 14 | c412530_g3_i2 | Fire ant venom protein (Sol i II) | 605 | 99 | 0 | 8.46E-09 | L09560 | 16.47 | 93.02 | 8.81 |
| 15 | c376628_g1_i1 | histone-lysine N-methyltransferase SETMAR-like | 872 | 98 | 0 | 5.39E-08 | 105144447 | 16.29 | 8.65 | 0.82 |
| 16 | c43453_g1_i1 | ELMO domain-containing protein 2 | 1879 | 100 | 8E-171 | 0.000273 | XM_011158456 | 16.14 | 3.79 | 0.33 |
| 17 | c402879_g1_i1 | THAP domain-containing protein 4-like |  |  |  | 0.002428 | 100570331 | 15.84 | 0.53 | 0.05 |
| 18 | c76047_g1_i1 | pikachurin | 12467 | 84 | 0 | 0.003595 | XM_012010396 | 14.41 | 0.47 | 0.04 |
| 19 | c44648_g1_i1 | CQMa 102 nitroreductase | 621 | 79 | 1E-65 | 0.003595 | XM_007816732 | 14.41 | 1.48 | 0.14 |
| 20 | c409381_g3_i1 | homeobox protein cut | 6012 | 91 | 2E-107 | 0.000776 | XM_012017737 | 13.85 | 4.36 | 0.44 |
| 21 | c404103_g2_i2 | tensin-1 | 6245 | 100 | 0 | 0.000276 | XM_011159718 | 13.64 | 0.93 | 0.10 |
| 22 | c440977_g1_i1 | myb-like protein A | 2341 | 100 | 0 | 1.91E-05 | XM_011166650 | 13.16 | 3.62 | 0.42 |
| 23 | c370960_g1_i2 | homeobox protein goosecoid-like | 733 | 100 | 5E-98 | 0.025273 | XM_011166088 | 12.98 | 2.24 | 0.23 |
| 24 | c435341_g1_i1 | CDK5RAP3-like protein | 1505 | 100 | 3E-16 | 0.025273 | XM_011168794 | 12.98 | 1.27 | 0.13 |
| 25 | c396407_g1_i2 | major facilitator superfamily domain-containing protein 6 | 2999 | 100 | 0 | 0.025273 | XM_011160948 | 12.98 | 0.62 | 0.07 |
| 26 | c407535_g3_i1 | AP-1 complex subunit gamma-1 | 3919 | 100 | 1E-96 | 0.025273 | XM_011173163 | 12.98 | 0.54 | 0.06 |
| 27 | c433470_g1_i1 | fatty acid hydroxylase domain-containing protein 2 | 2510 | 100 | 1E-27 | 0.025273 | XM_011159195 | 12.98 | 0.71 | 0.07 |
| 28 | c411609_g2_i1 | acetyl-coenzyme A transporter 1 | 2364 | 100 | 3E-150 | 2.48E-05 | XM_011164663 | 12.89 | 1.59 | 0.19 |
| 29 | c378275_g1_i1 | cadherin-87A | 6533 | 99 | 0 | 0.000424 | XM_011173091 | 12.59 | 1.08 | 0.13 |
| 30 | c407879_g1_i2 | multiple epidermal growth factor-like domains protein 10 | 1780 | 99 | 0 | 2.3E-05 | XM_011159318 | 12.47 | 0.90 | 0.11 |
| 31 | c408624_g1_i1 | Neff eukaryotic initiation factor 5a | 471 | 97 | 0 | 0.00136 | XM_004337894 | 12.32 | 1.29 | 0.15 |
| 32 | c83238_g1_i1 | homeobox protein extradenticle | 8132 | 85 | 2E-128 | 0.000528 | XM_012024551 | 12.07 | 2.27 | 0.28 |
| 33 | c411584_g4_i1 | guanine nucleotide-releasing factor 2-like | 640 | 100 | 9E-114 | 9.49E-06 | XM_011175730 | 11.90 | 3.57 | 0.46 |
| 34 | c173115_g2_i1 | mediator of RNA polymerase II transcription subunit 15 | 2817 | 100 | 3E-138 | 0.036674 | XM_011167316 | 11.55 | 0.50 | 0.06 |
| 35 | c373208_g1_i1 | protein still life, isoform SIF type 1 | 10279 | 100 | 2E-69 | 0.036674 | XM_011168143 | 11.55 | 0.75 | 0.09 |
| 36 | c394069_g2_i1 | nesprin-1 | 47187 | 99 | 0 | 0.00182 | XM_011177913 | 11.55 | 1.17 | 0.15 |
| 37 | c385540_g1_i1 | leucine-rich repeat serine/threonine-protein kinase 1 | 9008 | 100 | 0 | 0.000462 | XM_011171958 | 11.15 | 1.65 | 0.22 |
| 38 | c393634_g1_i1 | carboxypeptidase Q-like | 1721 | 99 | 1E-177 | 7.17E-06 | XM_011169463 | 11.00 | 4.14 | 0.58 |
| 39 | c158752_g1_i1 | trichohyalin-like |  |  |  | 0.003334 | 105679557 | 10.79 | 1.75 | 0.23 |
| 40 | c179799_g1_i1 | neurogenic locus Notch protein | 6043 | 100 | 0 | 0.003334 | XM_011170091 | 10.79 | 1.27 | 0.17 |
| 41 | c425973_g1_i1 | ELKS/Rab6-interacting/CAST family member 1-like | 1410 | 100 | 3E-132 | 0.003334 | XM_011158880 | 10.79 | 1.25 | 0.17 |
| 42 | c406205_g3_i1 | CUGBP Elav-like family member 4 | 5059 | 100 | 5E-22 | 0.001331 | XM_011347821 | 10.50 | 1.07 | 0.15 |
| 43 | c410396_g2_i1 | phosphate carrier protein, mitochondrial-like | 846 | 100 | 7E-120 | 0.001331 | XM_011161521 | 10.50 | 1.06 | 0.15 |
| 44 | c412327_g10_i1 | DNA replication licensing factor Mcm2-like | 2386 | 94 | 2E-12 | 9.26E-05 | XM_011177365 | 10.13 | 14.13 | 2.05 |
| 45 | c384682_g1_i1 | putative golgin subfamily A member 6-like protein 6 | 2574 | 100 | 0 | 0.004567 | 105667571 | 10.02 | 1.95 | 0.28 |
| 46 | c4198_g1_i1 | STE20-like serine/threonine-protein kinase | 797 | 100 | 0 | 0.004567 | XM_011161508 | 10.02 | 0.95 | 0.14 |
| 47 | c134197_g1_i1 | vigilin-like | 4236 | 100 | 3E-133 | 0.004567 | XM_011164504 | 10.02 | 0.77 | 0.11 |
| 48 | c412886_g4_i1 | fatty acyl-CoA reductase 1-like | 1173 | 90 | 1E-111 | 0.004567 | XM_011169612 | 10.02 | 0.82 | 0.12 |

Table S11.

| **No** | **Contig** | **Gene** | **Target length** | **Identites (%)** | **e-value** | **Corrected *P*-value** | **Gene bank accession number** | **Fold change** | FPKM | |
| --- | --- | --- | --- | --- | --- | --- | --- | --- | --- | --- |
|  |  |  |  |  |  |  |  |  | T30 | T40 |
| 1 | c412911_g4_i1 | nucleolar protein 14 homolog | 4654 | 89 | 5E-21 | 1.1895E-08 | XM_011163621 | 76.70 | 0.07 | 8.25 |
| 2 | c412971_g2_i1 | fatty acid synthase-like | 4696 | 83 | 0 | 2.09086E-11 | XM_011170571 | 72.48 | 0.12 | 12.79 |
| 3 | c408847_g1_i1 | fatty acid synthase-like | 1306 | 97 | 0 | 9.14813E-11 | XM_011176330 | 42.50 | 0.56 | 34.93 |
| 4 | c408760_g1_i1 | DEAD-box ATP-dependent RNA helicase 20-like | 3339 | 100 | 0 | 1.94657E-10 | XM_011170647 | 34.92 | 0.11 | 5.46 |
| 5 | c396544_g1_i2 | lachesin-like | 2185 | 99 | 0 | 8.90431E-06 | XM_011164366 | 34.85 | 0.03 | 1.40 |
| 6 | c399377_g1_i2 | transcription factor hamlet-like | 2282 | 99 | 0 | 8.10358E-08 | XM_011170316 | 30.88 | 0.04 | 1.73 |
| 7 | c410393_g9_i1 | odorant receptor 63a-like | 1754 | 100 | 0 | 7.4683E-08 | 105146572 | 26.81 | 0.17 | 6.67 |
| 8 | c412527_g9_i3 | high affinity cAMP-specific and IBMX-insensitive 3',5'-cyclic phosphodiesterase 8A | 3821 | 86 | 0 | 1.85518E-10 | XM_012675321 | 24.78 | 0.69 | 24.39 |
| 9 | c404183_g1_i2 | small glutamine-rich tetratricopeptide repeat-containing protein alpha-like | 2026 | 100 | 2E-59 | 4.92257E-06 | XM_011167035 | 24.22 | 0.13 | 4.82 |
| 10 | c399411_g1_i2 | mitochondrial coenzyme A transporter SLC25A42 | 1294 | 100 | 9E-49 | 0.00016724 | XM_011167831 | 23.79 | 0.06 | 2.22 |
| 11 | c412125_g8_i1 | microtubule-associated protein futsch | 7678 | 100 | 7E-167 | 9.60513E-07 | XM_011166138 | 23.52 | 0.08 | 2.63 |
| 12 | c93827_g1_i1 | protein Cep78 homolog | 1808 | 99 | 0 | 3.69893E-10 | XM_011160456 | 22.19 | 16.23 | 511.72 |
| 13 | c394437_g1_i1 | skin secretory protein xP2-like | 3971 | 80 | 0 | 4.36013E-07 | XM_012007292 | 21.91 | 0.07 | 2.14 |
| 14 | c412453_g1_i1 | 97 kDa heat shock protein | 4007 | 100 | 3E-48 | 2.03056E-08 | XM_011164210 | 21.83 | 0.17 | 5.21 |
| 15 | c376248_g1_i1 | mitochondrion | 16300 | 99 | 0 | 5.06947E-07 | KY018919 | 21.57 | 0.06 | 1.84 |
| 16 | c432326_g1_i1 | G protein alpha q subunit | 1579 | 98 | 8E-60 | 0.000272714 | XM_011163929 | 21.42 | 0.04 | 1.53 |
| 17 | c402976_g1_i3 | protein 5NUC-like | 1832 | 100 | 2E-79 | 0.000592165 | XM_011162950 | 19.06 | 0.06 | 1.99 |
| 18 | c406308_g1_i1 | high affinity cAMP-specific and IBMX-insensitive 3',5'-cyclic phosphodiesterase 8A | 3775 | 83 | 2E-115 | 4.18217E-09 | XM_012675160 | 19.04 | 0.59 | 16.05 |
| 19 | c406240_g1_i1 | proton-coupled folate transporter | 844 | 82 | 7E-37 | 0.000142643 | 105430822 | 18.92 | 0.23 | 6.36 |
| 20 | c378693_g1_i1 | RNA-binding protein fusilli | 3908 | 99 | 0 | 1.493E-05 | XM_011175061 | 16.73 | 0.12 | 2.93 |
| 21 | c401663_g2_i2 | eukaryotic peptide chain release factor subunit 1 | 1892 | 100 | 1E-116 | 8.3555E-05 | XM_011167694 | 16.72 | 0.13 | 3.33 |
| 22 | c412951_g3_i1 | protein dopey-1 homolog | 9748 | 100 | 4E-97 | 0.000101122 | XM_011158263 | 16.30 | 0.10 | 2.40 |
| 23 | c449847_g1_i1 | sestrin-3 | 6430 | 100 | 1E-143 | 6.38632E-06 | XM_011170797 | 16.10 | 0.95 | 23.82 |
| 24 | c378660_g1_i2 | mitochondrial DNA, complete sequence, strain: C57BL/6J | 16301 | 100 | 0 | 3.57577E-08 | AP014886 | 16.06 | 0.61 | 14.07 |
| 25 | c393414_g1_i1 | mitochondrion | 16300 | 100 | 0 | 3.33182E-08 | KY018919 | 15.96 | 0.78 | 17.77 |
| 26 | c455967_g1_i1 | tetratricopeptide repeat protein 21B-like | 4218 | 100 | 0 | 0.00182029 | XM_011170879 | 15.90 | 0.04 | 1.10 |
| 27 | c403566_g1_i1 | voltage-dependent L-type calcium channel subunit beta-2 | 3363 | 85 | 7E-49 | 0.002453872 | XR_001009484 | 15.10 | 0.08 | 1.87 |
| 28 | c401880_g1_i1 | procollagen-lysine,2-oxoglutarate 5-dioxygenase 1 | 2514 | 100 | 0 | 4.19849E-05 | 105620057 | 14.75 | 0.13 | 2.83 |
| 29 | c416194_g1_i1 | PHD finger protein rhinoceros | 11218 | 100 | 0 | 0.003334058 | XM_011173082 | 14.32 | 0.05 | 1.17 |
| 30 | c402818_g2_i1 | phospholipase A1-like | 1148 | 100 | 5E-98 | 0.000276252 | XM_011175277 | 14.22 | 0.07 | 1.50 |
| 31 | c415368_g1_i1 | RNA-binding protein fusilli | 5526 | 81 | 4E-31 | 5.74449E-05 | XM_012379554 | 14.19 | 0.24 | 5.03 |
| 32 | c397432_g1_i2 | SRSF protein kinase 3 | 3736 | 96 | 0 | 0.000341486 | XM_011157893 | 13.80 | 0.14 | 2.85 |
| 33 | c411764_g2_i1 | fatty acid synthase-like | 3512 | 98 | 1E-22 | 8.94946E-07 | XM_011174609 | 13.79 | 0.84 | 16.91 |
| 34 | c446889_g1_i1 | probable basic-leucine zipper transcription factor Q | 2761 | 100 | 0 | 4.55536E-07 | XM_011169146 | 13.77 | 0.39 | 7.75 |
| 35 | c399334_g2_i2 | four and a half LIM domains protein 2 | 1752 | 82 | 6E-46 | 2.04188E-06 | XM_018493959 | 13.70 | 0.48 | 9.51 |
| 36 | c383558_g1_i2 | fructose-bisphosphate aldolase-like | 1275 | 100 | 2E-164 | 7.92958E-05 | XM_011165105 | 13.62 | 0.17 | 3.37 |
| 37 | c405998_g1_i1 | headcase protein | 5089 | 99 | 0 | 1.30044E-05 | XM_011167755 | 13.47 | 0.17 | 3.33 |
| 38 | c405270_g2_i1 | protein diaphanous | 6031 | 100 | 0 | 9.34878E-05 | XM_011171339 | 13.34 | 0.20 | 3.91 |
| 39 | c406386_g1_i1 | fatty acid synthase-like | 976 | 98 | 6E-137 | 1.62783E-05 | XM_011176452 | 12.95 | 0.87 | 17.10 |
| 40 | c201974_g1_i1 | protein broad-minded-like | 3583 | 99 | 0 | 1.82431E-05 | XM_011177465 | 12.78 | 0.09 | 1.59 |
| 41 | c334230_g1_i1 | protein Tob1 | 8436 | 100 | 0 | 0.006310965 | XM_011164427 | 12.73 | 0.09 | 1.92 |
| 42 | c372918_g1_i1 | baculoviral IAP repeat-containing protein 6 | 15789 | 100 | 0 | 0.006310965 | XM_011174420 | 12.74 | 0.05 | 1.01 |
| 43 | c402147_g1_i2 | rho GDP-dissociation inhibitor 2 | 2496 | 79 | 3E-95 | 4.22924E-05 | XM_018501106 | 12.67 | 0.12 | 2.22 |
| 44 | c405803_g1_i1 | tubulin polyglutamylase | 1690 | 99 | 0 | 1.33783E-05 | XM_011172479 | 12.66 | 0.09 | 1.61 |
| 45 | c407496_g1_i1 | trypsin-1 | 1999 | 99 | 0 | 1.46199E-06 | XM_011158593 | 12.55 | 0.21 | 3.75 |
| 46 | c407533_g1_i2 | trinucleotide repeat-containing gene 6C protein | 15455 | 100 | 0 | 4.85467E-05 | XM_011162233 | 12.45 | 0.13 | 2.43 |
| 47 | c410379_g1_i1 | transposon mariner | 1382 | 90 | 0 | 5.58196E-05 | AF518173 | 12.24 | 0.17 | 3.06 |
| 48 | c408179_g1_i2 | circadian clock-controlled protein-like | 1262 | 99 | 0 | 1.77048E-07 | XM_011172747 | 12.23 | 4.65 | 81.23 |
| 49 | c401789_g2_i1 | BAG domain-containing protein Samui-like | 5065 | 100 | 4E-136 | 9.33769E-07 | XM_011170865 | 11.96 | 0.47 | 8.03 |
| 50 | c401040_g1_i3 | putative nuclease HARBI1 | 645 | 99 | 0 | 4.74108E-05 | XM_011172189 | 11.40 | 0.11 | 1.78 |
| 51 | c413244_g1_i3 | multidrug resistance-associated protein 4-like | 2937 | 99 | 0 | 3.11528E-07 | XM_011169881 | 11.36 | 2.42 | 39.03 |
| 52 | c455177_g1_i1 | eye-specific diacylglycerol kinase | 5386 | 100 | 0 | 0.001048998 | XM_011158051 | 11.30 | 0.07 | 1.25 |
| 53 | c404729_g1_i1 | polypeptide N-acetylgalactosaminyltransferase 2 | 2723 | 91 | 1E-126 | 0.012384085 | XM_012677796 | 11.16 | 0.06 | 1.14 |
| 54 | c410448_g1_i7 | tyrosine aminotransferase | 1695 | 99 | 0 | 1.54539E-06 | XM_011163521 | 11.14 | 0.45 | 7.17 |
| 55 | c438991_g1_i1 | beta-1,3-galactosyltransferase 5 | 1967 | 100 | 0 | 0.000317778 | XM_011170444 | 11.07 | 0.15 | 2.45 |
| 56 | c394396_g1_i1 | fatty acid synthase-like | 6347 | 84 | 1E-78 | 6.93079E-05 | XM_011165759 | 10.88 | 0.93 | 15.41 |
| 57 | c164659_g1_i1 | heat shock protein 83 | 2616 | 99 | 0 | 4.66627E-07 | XM_011173022 | 10.75 | 80.97 | 1238.96 |
| 58 | c409955_g3_i2 | sialin-like | 2727 | 100 | 1E-41 | 1.63962E-05 | XM_011177863 | 10.73 | 0.22 | 3.36 |
| 59 | c438745_g1_i1 | ejaculatory bulb-specific protein 3 | 605 | 100 | 0 | 6.2349E-07 | XM_011161971 | 10.53 | 26.80 | 406.61 |
| 60 | c115317_g1_i1 | S-adenosylmethionine decarboxylase proenzyme | 3985 | 100 | 2E-92 | 0.001696707 | XM_011170856 | 10.47 | 0.23 | 3.73 |
| 61 | c373218_g1_i1 | glycogen-binding subunit 76A | 4004 | 99 | 0 | 6.3892E-05 | XM_011157136 | 10.43 | 0.16 | 2.34 |
| 62 | c402693_g1_i1 | RB1-inducible coiled-coil protein 1 | 6456 | 92 | 8E-51 | 0.017601561 | XM_011631686 | 10.37 | 0.26 | 4.46 |
| 63 | c404366_g1_i1 | facilitated trehalose transporter Tret1-2 homolog | 2567 | 79 | 9E-111 | 8.99122E-05 | XM_012684887 | 10.37 | 0.14 | 2.07 |
| 64 | c355199_g2_i1 | elongation of very long chain fatty acids protein AAEL008004-like | 3862 | 90 | 6E-15 | 2.08373E-06 | XM_011692183 | 10.20 | 3.39 | 50.26 |
| 65 | c412347_g3_i1 | activator of 90 kDa heat shock protein ATPase homolog 1 | 2037 | 99 | 0 | 9.2711E-07 | XM_011166498 | 10.12 | 5.06 | 72.88 |
| 66 | c398012_g1_i1 | neprilysin-1-like | 2474 | 100 | 0 | 0.002174129 | 105202797 | 10.12 | 0.11 | 1.64 |

Table S12.

| **Contig** | **Unigene** | **Target length** | **Identities (%)** | **e-value** | **KEGG number** | **Enriched KEGG pathways** |
| --- | --- | --- | --- | --- | --- | --- |
| **T10/T30** | | | | | | |
| c409037_g1_i1 | pyruvate carboxylase | 5840 | 99 | 0 | 105192780 | Metabolic pathway |
|  |  |  |  |  |  | Pyruvate metabolism |
|  |  |  |  |  |  | Carbon metabolism |
|  |  |  |  |  |  | Citrate cycle (TCA cycle) |
|  |  |  |  |  |  | Biosynthesis of amino acids |
| c375063_g1_i1 | dual oxidase | 4819 | 99 | 0 | 105193293 | Toll and Imd signaling pathway |
|  |  |  |  |  |  | MAPK signaling pathway |
| c92359_g1_i1 | presenilin-1 | 1914 | 100 | 3E-119 | 105196124 | Wnt signaling pathway |
|  |  |  |  |  |  | Notch signaling pathway |
| c410285_g1_i1 | CD63 antigen-like | 1672 | 100 | 4E-110 | 105203107 | Lysosome |
| c406600_g3_i2 | actin | 3254 | 100 | 0 | 105201514 | Hippo signaling pathway - fly |
|  |  |  |  |  |  | Phagosome |
|  |  |  |  |  |  | Phototransduction - fly |
| c405878_g2_i1 | laminin subunit alpha-1 | 13963 | 84 | 0 | 105193731 | ECM-receptor interaction |
| c396785_g2_i1 | nucleoporin p54 | 2108 | 100 | 0 | 105202099 | RNA transport |
| c372556_g1_i1 | diacylglycerol kinase 1 | 4296 | 100 | 0 | 105199906 | Glycerophospholipid metabolism |
|  |  |  |  |  |  | Glycerolipid metabolism |
|  |  |  |  |  |  | Phosphatidylinositol signaling system |
|  |  |  |  |  |  | Metabolic pathway |
| c405044_g1_i1 | translation initiation factor 3 | 2593 | 100 | 1E-66 | 105208146 | RNA transport |
| c400319_g1_i1 | hexaprenyldihydroxybenzoate methyltransferase | 1617 | 99 | 0 | 105195087 | Ubiquinone and other terpenoid-quinone biosynthesis |
|  |  |  |  |  |  | Metabolic pathway |
| c408362_g1_i1 | U6 snRNA-associated Sm-like protein LSm4 | 1061 | 100 | 0 | 105199619 | Spliceosome |
|  |  |  |  |  |  | RNA degradation |
| c389696_g1_i1 | protein expanded | 6525 | 99 | 0 | 105199061 | Hippo signaling pathway - multiple species |
|  |  |  |  |  |  | Hippo signaling pathway - fly |
| c415378_g1_i1 | sphingomyelin phosphodiesterase 1-like | 1885 | 100 | 2E-180 | 105205844 | Metabolic pathways |
|  |  |  |  |  |  | Lysosome |
|  |  |  |  |  |  | Sphingolipid metabolism |
| c371411_g1_i1 | proteasome non-ATPase regulatory | 5533 | 100 | 2E-148 | 105197079 | Proteasome |
| c409005_g1_i2 | transcription initiation factor IIA | 2422 | 100 | 0 | 105197424 | Basal transcription factors |
| c431944_g1_i1 | adenylate cyclase type 6 | 5660 | 99 | 8E-171 | 105204551 | Metabolic pathways |
|  |  |  |  |  |  | Purine metabolism |
|  |  |  |  |  |  | Longevity regulating pathway - multiple species |
| c172501_g1_i1 | thioredoxin domain-containing protein 5 | 1833 | 100 | 0 | 105195709 | Protein processing in endoplasmic reticulum |
| c356244_g1_i1 | ADP-dependent glucokinase | 1937 | 100 | 0 | 105205417 | Metabolic pathways |
|  |  |  |  |  |  | Glycolysis / Gluconeogenesis |
|  |  |  |  |  |  | Carbon metabolism |
| c393714_g1_i1 | DNA mismatch repair protein Msh6 | 3093 | 99 | 0 | 105205867 | Mismatch repair |
| c417775_g1_i1 | E2/E3 hybrid ubiquitin-protein ligase UBE2O | 4735 | 100 | 2E-83 | 105198011 | Ubiquitin mediated proteolysis |
| c5572_g1_i1 | porphobilinogen deaminase-like | 1733 | 100 | 7E-165 | 105203963 | Metabolic pathways |
|  |  |  |  |  |  | Porphyrin and chlorophyll metabolism |
| c412839_g2_i1 | glucose-regulated protein (HSP70) | 1986 | 91 | 0 | MYCTH_2315513 | Protein export |
|  |  |  |  |  |  | Protein processing in endoplasmic reticulum |
| **T20/T30** | | | | | | |
| c412852_g4_i1 | DNA- (apurinic or apyrimidinic site) lyase | 398 | 82 | 0.0003 | 105193701 | Base excision repair |
| c411702_g1_i3 | muscle M-line assembly protein unc-89 | 9837 | 100 | 0 | 105193639 | Glycerophospholipid metabolism |
|  |  |  |  |  |  | Metabolic pathways |
| c376628_g1_i1 | histone-lysine N-methyltransferase SETMAR-like | 245 | 49 | 2E-09 | 105144447 | Metabolic pathways |
|  |  |  |  |  |  | Lysine degradation |
| c407535_g3_i1 | AP-1 complex subunit gamma-1 | 3919 | 100 | 1E-96 | 105204102 | Lysosome |
| c411609_g2_i1 | acetyl-coenzyme A transporter 1 | 2364 | 100 | 3E-150 | 105198028 | Metabolic pathways |
|  |  |  |  |  |  | Glycosphingolipid biosynthesis-ganglio series |
| **T40/T30** | | | | | | |
| c412971_g2_i1 | fatty acid synthase | 4696 | 83 | 0 | 105151862 | Fatty acid synthase |
|  |  |  |  |  |  | Metabolic pathway |
|  |  |  |  |  |  | Fatty acid metabolism |
| c408760_g1_i1 | DEAD-box ATP-dependent RNA helicase 20-like | 3339 | 100 | 0 | 105202237 | Spliceosome |
| c412527_g9_i3 | phosphodiesterase 8A | 3821 | 86 | 0 | 105195169 | Purine metabolism |
|  |  |  |  |  |  | Metabolic pathways |
| c412453_g1_i1 | heat shock 70 kDa protein 4L isoform X1 | 4007 | 100 | 3E-48 | 105251276 | Protein processing in endoplasmic reticulum |
| c432326_g1_i1 | G protein alpha q subunit | 1579 | 98 | 8E-60 | 105197512 | Phototransduction-fly |
| c401880_g1_i1 | procollagen-lysine,2-oxoglutarate 5-dioxygenase 1 | 112 | 65 | 3E-23 | 105620057 | Lysine degradation |
|  |  |  |  |  |  | Metabolic pathways |
| c411764_g2_i1 | fatty acid synthase-like | 3512 | 98 | 1E-22 | 105205264 | Fatty acid synthase |
|  |  |  |  |  |  | Metabolic pathway |
|  |  |  |  |  |  | Fatty acid metabolism |
| c383558_g1_i2 | fructose-bisphosphate aldolase-like | 1275 | 100 | 2E-164 | 105198405 | Glycolysis/Gluconeogenesis |
|  |  |  |  |  |  | Pentose phosphate pathway |
|  |  |  |  |  |  | Fructose and mannose metabolism |
|  |  |  |  |  |  | Metabolic pathways |
|  |  |  |  |  |  | Carbon metabolism |
|  |  |  |  |  |  | Biosynthesis of amino acids |
| c402147_g1_i2 | histone-lysine N-methyltransferase SETMAR-like | 2496 | 79 | 3E-95 | 105433874 | Lysine degradation |
|  |  |  |  |  |  | Metabolic pathways |
| c413244_g1_i3 | multidrug resistance-associated protein 4-like | 2937 | 99 | 0 | 105153301 | ABC transporters |
| c455177_g1_i1 | eye-specific diacylglycerol kinase | 5386 | 100 | 0 | 105193549 | Glycerolipid metabolism |
|  |  |  |  |  |  | Glycerophospholipid metabolism |
|  |  |  |  |  |  | Metabolic pathways |
|  |  |  |  |  |  | Phosphatidylinositol signaling system |
| c410448_g1_i7 | tyrosine aminotransferase | 1695 | 99 | 0 | 105197229 | Ubiquinone and other terpenoid-quinone biosynthesis |
|  |  |  |  |  |  | Cysteine and methionine metabolism |
|  |  |  |  |  |  | Tyrosine metabolism |
|  |  |  |  |  |  | Phenylalanine metabolism |
|  |  |  |  |  |  | Phenylalanine, tyrosine and tryptophan biosynthesis |
|  |  |  |  |  |  | Metabolic pathways |
|  |  |  |  |  |  | Biosynthesis of amino acids |
| c438991_g1_i1 | beta-1,3-galactosyltransferase 5 | 1967 | 100 | 0 | 105202068 | Metabolic pathways |
|  |  |  |  |  |  | Glycosphingolipid biosynthesis-lacto and neolactoseries |
| c164659_g1_i1 | heat shock protein 83 | 2616 | 99 | 0 | 105204027 | Protein processing in endoplasmic reticulum |
| c115317_g1_i1 | S-adenosylmethionine decarboxylase proenzyme | 3985 | 100 | 2E-92 | 105202377 | Cycteine and methionine metabolism |
|  |  |  |  |  |  | Arginine and proline metabolism |
|  |  |  |  |  |  | Metabolic pathways |

Table S13.

| Primer | Use | Orientation | Sequence (5ʹ - 3ʹ) | Annealing  temperature (°C) | Amplicon  (bp) |
| --- | --- | --- | --- | --- | --- |
| Si_GPDH | qPCR | Forward  Reverse | CCACACACTTCCACAGTATCA  ATGTAAGGACAAGCGACTGG | 52 | 102 |
| Si-hsp70 | qPCR | Forward  Reverse | TCTCAACGTCCTCCGTATCA  AAGGAGCGACACATCGAAAG | 52 | 133 |
| Si-synapsin | qPCR | Forward  Reverse | CGACCAGGGTGCTTCTATTT  ATTTGCTCCAGTCGGTGTT | 52 | 125 |
| Si-cuticle | qPCR | Forward  Reverse | ATTGTCGCGTTCGTACATACA  GATAGACAGGAGAACGGAGAGA | 52 | 95 |
| Si-fatty acid synthase | qPCR | Forward  Reverse | ATCATTACCACCAACGGTAACT  GTTGCGCACCAGCATTATAC | 52 | 110 |
| Si-cytochrome P450 6K1 | qPCR | Forward  Reverse | AACGCTTTCCTCTCAGGTATTT  ATGACCTCCTTACCCTATCCTC | 52 | 96 |
| Si-serine protease | qPCR | Forward  Reverse | CGACCTATGGACAGGAAATCAG  GGTAAACTTGCAGGCAATCATC | 52 | 90 |
| Si-ef1β | qPCR | Forward  Reverse | TGAAGACCGATAAGGGCA  TCGTCCGAACCAAAGAGA | 52 | 300 |
| Si-lipase | qPCR | Forward  Reverse | AGTCCACGAATGTCGAACATAAA  CAACTCGCCTTGGAGTCATC | 52 | 115 |
| Si-trypsin | qPCR | Forward  Reverse | GAGATCCGTCATCGGTGTTAAT  GTGATGCTGCGGATGTATCT | 52 | 91 |
| Si-chymotrypsin | qPCR | Forward  Reverse | ACAGCTGGACATTGCATTAAAC  CACGAGGCGTGTCAAGATAA | 52 | 82 |
| Si-transmembrane channel like-protein | qPCR | Forward  Reverse | GCAGGTCCGTATCATGGAAATA  CAGAAGAGCCAACGGAGAAA | 52 | 81 |
| Si-calmodulin | qPCR | Forward  Reverse | TGAGAATTCATCGTCTGTGAAA  GACTTCTACGGTCGACTAAATAAA | 52 | 133 |
| Si-monocarboxylate transporter | qPCR | Forward  Reverse | CTGTCGTCGGGCATGTATATC  CGAACAACGATATCCCGTAAGA | 52 | 97 |
| Si-general odorant-binding protein | qPCR | Forward  Reverse | TGAAAGCAGTTGCGACTAAATG GTGATCTGGATCCGTGGAATAA | 52 | 104 |
| Si-small G-protein | qPCR | Forward  Reverse | TGTGCTTCTCCATACCCAATATC GCTAATGCGTGTTTCAGTCATC | 52 | 107 |
| Si-RNA binding protein | qPCR | Forward  Reverse | GGTCAGGGTGACAAGGAAAT AGGTAGAGTCGTCGATGGAA | 52 | 139 |
| Si-dipeptidase | qPCR | Forward  Reverse | CGCAGGAACTCATGGAAGATAG GAAACGGTTGCACGAACAATAG | 52 | 92 |
| Si-fibrinogen silencer-binding protein | qPCR | Forward  Reverse | AACGTATACTTCGTGCGTGTTA TTACCCAACCTTTCCCTCTTTC | 52 | 101 |
| Si-anoctamin | qPCR | Forward  Reverse | CCTGCCCAGAGATCGTTAATAG GAGATACGTTCCATCGGCTAAA | 52 | 108 |
| Si-scavenger receptor | qPCR | Forward  Revers | GCTAATCTCCAGGAAGGGAAAG CGGTATGCTACTGAGCACTATG | 52 | 100 |
